# Impact of Varying Velocities and Solvation Boxes on Alchemical Free-Energy Simulations

**DOI:** 10.1021/acs.jcim.4c02236

**Published:** 2025-01-31

**Authors:** Meiting Wang, Hao Jiang, Ulf Ryde

**Affiliations:** † School of Medical Engineering & Xinxiang Key Laboratory of Biomedical Information Research & Henan International Joint Laboratory of Neural Information Analysis and Drug Intelligent Design & Xinxiang Key Laboratory of Biomedical Information Research, Xinxiang Medical University, Xinxiang 453003, China; ‡ Department of Computational Chemistry, Lund University, Chemical Centre, P.O. Box 124, Lund SE-221 00, Sweden

## Abstract

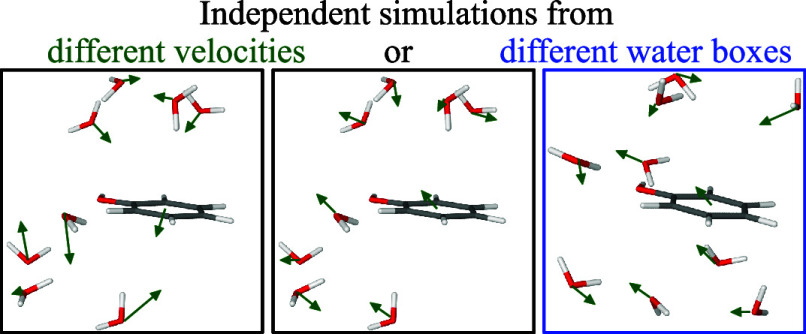

Alchemical free-energy perturbation (FEP) is an accurate and thermodynamically stringent way to estimate relative energies for the binding of small ligands to biological macromolecules. It has repeatedly been pointed out that a single simulation normally stays near the starting point in phase space and therefore underestimates the uncertainty of the results. Therefore, it is better to run an ensemble of independent simulations. Traditionally, such an ensemble has been generated by using different starting velocities. We argue that it is better to use also other random choices made during the setup of the simulations, in particular the solvation of the solute. We show here that such solvent-induced independent simulations (SIS) sometimes give a larger standard deviation and slightly different results for the binding of 42 ligands to five different proteins, viz. human N-terminal bromodomain 4, the Leu99Ala mutant of T4 lysozyme, dihydrofolate reductase, blood-clotting factor Xa, and ferritin. SIS does not involve any increase in the time consumption. Therefore, we strongly recommend the use of SIS (in addition to different velocities) to start independent simulations. Other random or uncertain choices in the setup of the simulated systems, e.g., the selection of residues with alternative conformations or positions of added protons, may also be used to enhance the variation in independent simulations.

## Introduction

A major goal of computational biochemistry is to calculate the free energy for the binding of small molecules to biological macromolecules.^1,2^ In particular, this is of central interest in drug development. Consequently, many computational methods have been developed with this aim,^1^ ranging from molecular docking and scoring,^3,4^ via simulation-based methods like MM/PBSA, MM/GBSA (molecular mechanics combined with Poisson–Boltzmann or generalized Born and surface-area solvation)^5−7^ and linear interaction energy,^8,9^ to alchemical free-energy perturbation (FEP)^10−12^ and nonequilibrium approaches.^13,14^ In the FEP methods, a ligand is slowly converted to another ligand or to a noninteracting molecule during molecular-dynamics (MD) or Monte Carlo simulations and the free energies are calculated by methods like the exponential averaging,^15^ thermodynamic integration,^16^ Bennett acceptance ratio (BAR)^17^ or multistate BAR (MBAR).^18^ Several studies have shown that FEP approaches can reach an accuracy of 4 kJ/mol or better for well-behaving systems.^19−24^ FEP methods are based on an exact statistical-mechanics framework and therefore, the accuracy is limited only by the accuracy of the force field and the extent of phase-space sampling.^10−12^

MD simulations are very sensitive to the simulation parameters. A small perturbation of the simulation will grow exponentially with the simulation time (the Lyapunov instability).^25^ The simulations depend on many types of factors, e.g., the random seed, program parameters and force-field parameters.^26,27^ It has repeatedly been pointed out that the phase-space sampling of MD simulations is rather slow, so that the system stays close to the starting state and results obtained with limited sampling depend on the starting conditions.^28−32^ In particular, the precision of binding free energies estimated from a single simulation typically underestimates the true uncertainty.^33−40^ Consequently, it has been recommended that several independent simulations with different starting conditions should be run and that the uncertainty should be estimated from the spread of the results from these simulations.^33,34,36,37,39−41^ In particular, it has been shown that such an approach is more efficient that running a single FEP simulation for longer time.^28,31,33,34,41−44^

Independent simulations are typically generated by employing different random seeds when assigning the starting velocities of the MD simulations.^28,29,31,32,37,40,44^ MD simulations are normally started from a crystal structure with a cocrystallized or a docked ligand, providing coordinates for the macromolecule, the ligand and some solvent molecules. However, the velocities need also to be specified when starting MD simulations and these are completely unknown. Therefore, they are normally assigned random values and directions, following a Boltzmann distribution. Therefore, it is natural and simple to exploit this randomness when constructing an ensemble of independent simulations. By employing the starting time when constructing the random seed, such a procedure can be made completely automatic.

However, there are also other parts of the setup of a MD simulation that involve random or at least highly arbitrary or uncertain steps.^34^ In particular, the crystal or docked structures contains only a limited number of solvent molecules and therefore many additional solvent molecules need to be added before the MD simulation can be started. The standard approach is to overlay the solute with an equilibrated periodic box of solvent molecules, taken from a equilibrated MD simulation of the pure solvent, removing solvent molecules that clash with the solute.^45^ Naturally, this is a very crude approach, because it gives no proper hydrogen bonds between the solute and solvent, and the solvent structure needs to relax and shrink toward the solute during the initial equilibration of the starting structure. Moreover, the approach is completely arbitrary in the meaning that any snapshot from an equilibrated solvent simulation would apply equally well and any rotation or translation of the solvent box or the protein would produce different results (in terms of both the solvent positions and the number of solvent molecules).

Therefore, it seems natural to employ also this uncertainty when setting up independent MD simulations. In this study, we compare these two approaches to generate independent simulations. As test cases, we calculate relative binding free energies of 42 ligands binding to five different proteins, viz. the human N-terminal bromodomain (BRD4),^38^ Leu99Ala mutant of bacteriophage T4 lysozyme,^46^ dihydrofolate reductase,^47,48^ blood-clotting factor Xa^49^ and ferritin.^50^ We compare the relative binding affinities and the precision obtained with the two approaches.

## Theory and Methods

### Simulation Details

**Figure 1:**
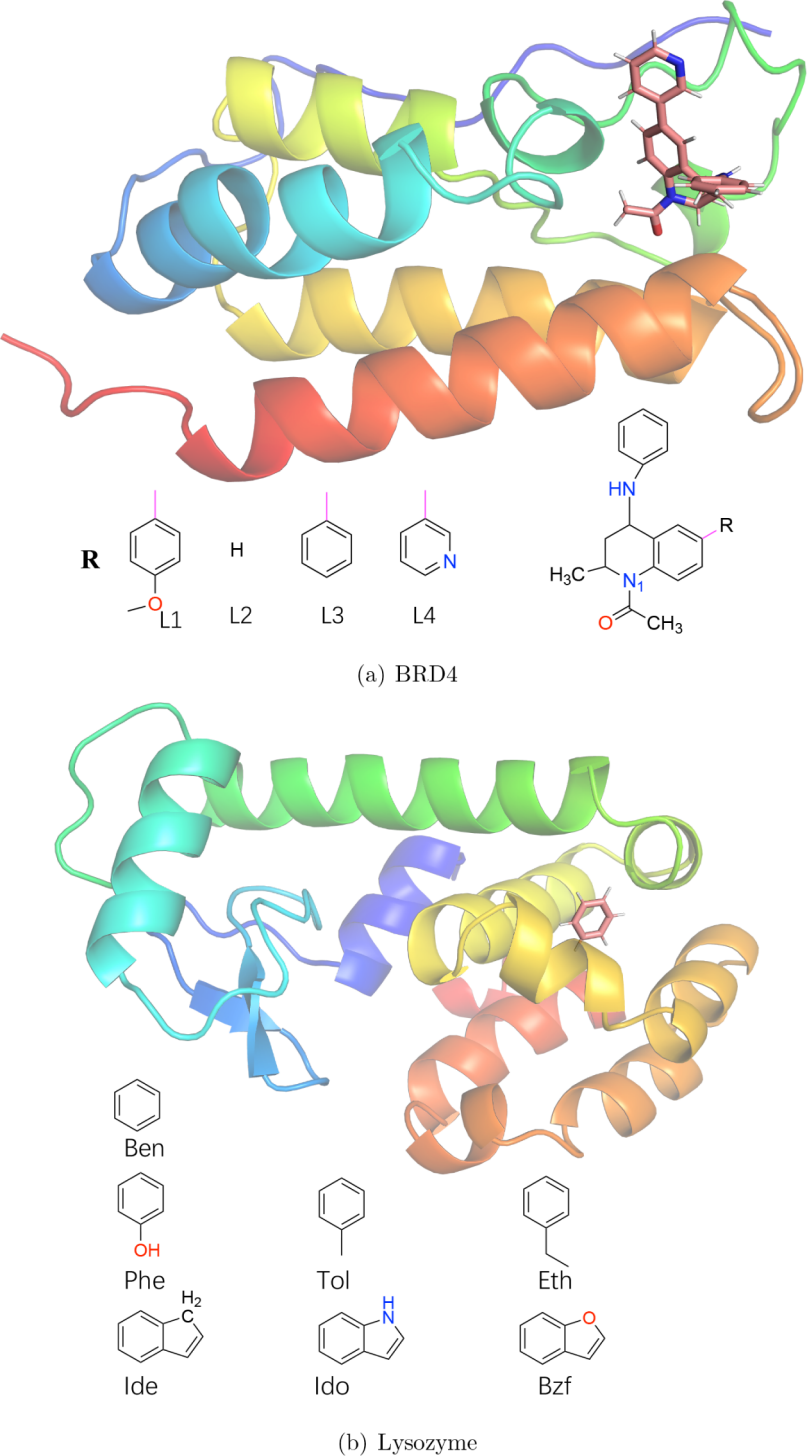
Schematic structures of the two proteins and structure formulas of the ligands. For the BRD4 ligands, all ligands have the same general scaffold (right) and only the R-group is varied (left; the pink line shows where the ring systems are connected to the scaffold).

We have calculated relative binding free energies of four ligands binding to BRD4^38^ and seven ligands binding to the Leu99Ala mutant of bacteriophage T4 lysozyme^46^ (the proteins and the ligands are shown in Figure 1). The simulations were based on the protein databank structures 4BJX (BRD4) and 181L (lysozyme). We employed the protein preparation wizard in Maestro software^51^ to add protons and flip side chains of Asn and Gln residues in the two proteins. All Arg, Lys, Asp and Glu residues were assumed charged. The protonation of the His residues was determined by considering the surrounding environment and hydrogen-bond network. There is only one His residue in each of the two proteins and our investigations suggest that both are protonated only on the NE2 atom. The ligands were docked into the proteins using Molecular Operating Environment (MOE2019).^52^

All simulations in this study were carried out with the AMBER 22 software.^45,53^ The proteins were modeled with the AMBER ff14SB force field,^54^ while the ligands were described by the GAFF2 general AMBER force field.^55^ Partial charges for the ligands were obtained with the AM1-BCC method using Antechamber, after optimization with the semiempirical AM1 method.^56^ Charges and parameters for the ligands are given in the Supporting Information. The protein–ligand complexes and the free ligands were solvated in a cubic periodic box of TIP3P water molecules extending at least 12 Å from any solute atom.^57^ Neutralizing counterions, sodium or chloride, were added to each protein using parameters from Joung and Cheatham.^55,58^

All systems were first minimized, employing 5000 cycles of steepest-descent and 5000 cycles of conjugate-gradient minimization without applying any restrains. Subsequently, a 50 ps heating at constant volume was performed, followed by 200 ps of equilibration at constant pressure. The temperature was controlled using a Langevin thermostat^59^ set to a target temperature of 300 K. Pressure regulation was achieved through a Monte Carlo barostat, maintaining a target pressure of 1.0 atm with a relaxation time of 2.0 ps.

During the FEP simulations, a ligand was gradually converted to another ligand in a transformation controlled by the transformation parameter λ.^60^ To ensure proper phase-space overlap, 11 alchemical windows (λ = 0.0, 0.1, 0.2, 0.3, 0.4, 0.5, 0.6, 0.7, 0.8, 0.9, 1.0) were used for the transformation and simulations at all λ were run simultaneously using the same starting structure. The nonbonded interactions were smoothly modified utilizing a soft-core potential^60^ involving both electrostatics and Lennard−Jones interactions. We employed the dual-topology approach implemented in AMBER^60^ and the simulations were run with the GPU version of the pmemd software.^62,63^ A 10 ns production was carried for each window in the NPT ensemble. The time step was set to 2 fs, and snapshots were saved every 2 ps for subsequent analysis. Particle mesh Ewald^64^ summation was used for electrostatic interactions and a cutoff of 8.0 Å was utilized for Lennard−Jones interactions.

We studied the effect of the starting velocities and the starting coordinates of the solvation water molecules on the relative binding free energies. We used five different sets of random starting velocities (obtained by using different random seeds, using the ig = −1 option in AMBER), as well as five different water boxes. These five distinct water configurations were taken from a 500 ns simulation of a TIP3P water box, taking snapshots every 12.5 ns.^34^ The water boxes are available at http://signe.teokem.lu.se/ulf/Methods/waterboxes.html (we used water boxes 6, 8, 16, 26 and 36). It should be noted that in simulations with different water boxes, the starting velocities were also randomized. Thus, a total of 10 independent FEP simulations were performed for each transformation.

To obtain more data, we also studied the binding of nine small substituted benzene ligands to a ferritin dimer (ten transformations),^50^ ten ligands to blood coagulation factor Xa (fXa) (ten transformations),^49^ and 12 ligands to dihydrofolate reductase (DHFR; 15 transformations).^47,48^ The ligands are shown in Figure S1 and are described in Table S1. The setup is described in our previous publications.^20,65^ The simulations differ in that 13 λ values were used (0.0, 0.05, 0.1, 0.2, 0.3, 0.4, 0.5, 0.6, 0.7, 0.8, 0.9, 0.95 and 1.0) and that the production run was only 2 ns.

### Free-Energy Estimators

FEP methods enable the calculation of relative free energy through a computationally efficient pathway. Using a standard thermodynamic cycle and the fact that the free energy is a state function, the relative binding free energy (ΔΔ*G*_bind_) between two ligands (L1 and L2) equals the difference between the absolute binding free energy (Δ*G*_bind_) of the two ligands, which involves changing the environment from unbound in an aqueous phase to bound in a complex with the protein target.^10−12^
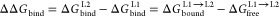
1

Here,  is the free energy difference for the ligands bound to the protein and  is the same free energy difference in water solution.

The free energy changes were estimated by MBAR method,^18^ as implemented in alchemlyb^66^ (https://alchemlyb.readthedocs.io). Moreover, we employed the Wu and Kofke bias measure Π to ensure that there is a proper overlap between all λ states.^67^ If Π < 0.5, additional λ values were added.

The performance of the free-energy estimates was quantified by the mean absolute deviation (MAD), the maximum error (Max), the correlation coefficient (*R*^2^) and Kendall’s rank correlation coefficient (τ) compared to experimental data. τ was calculated only for the transformations that were explicitly studied, not for all combinations that can be formed from these transformations (this is marked by calling it τ_*r*_). Moreover, it was evaluated considering only differences (both experimental and calculated) that are statistically significant at the 90% level (τ_*r*90_).^68^ Note that *R*^2^ for relative affinities depend on the direction of the perturbation (i.e., whether L1 → L2 or L2 → L1 is considered, which is arbitrary). This was solved by considering both directions (both forward and backward) for all perturbations when *R*^2^ was calculated.

The standard deviation of the quality measures was obtained by a simple simulation approach.^33^ For each transformation, 1000 Gaussian-distributed random numbers were generated with the mean and standard deviation equal to the MBAR and experimental results for that transformation. Then, the quality measures were calculated for each of these 1000 sets of simulated results and the standard error over the 1000 sets is reported as the uncertainty. Of course, such uncertainty estimates are quite approximate and assume a Gaussian distribution of the quality measures.

## Results and Discussion

In this study, we compare two approaches to obtain independent estimates for the calculation of relative binding free energies. In the first approach, five independent simulations were obtained by using a different random seed to generate the starting velocity. In the second approach, the proteins were solvated in five different solvent boxes. In the following, we will call these two approaches velocity-induced independent simulations (VIS) and solvent-induced independent simulations (SIS). We have studied five different proteins, bromodomain 4 (BRD4), the Leu99Ala mutant of T4 lysozyme, dihydrofolate reductase, blood-clotting factor Xa and ferritin. The results of the first two are described in detail in the main text, whereas the other three are described mainly in the Supporting Information.

For BRD4, we studied four relative affinities involving the four ligands shown in Figure 1. The results are collected in Table 1. It can be seen that the VIS and SIS approaches give quite similar results. The average ΔΔ*G*s differ by only 0.1–0.8 kJ/mol, which is within the statistical uncertainty–in no case is the difference larger than two times the precision (i.e., a 95% confidence *t* factor). Compared to the experimental estimates,^38^ the results are excellent, with errors of 0.1–1.8 kJ/mol and perfect both *R*^2^ and τ_*r*90_. There is no indication that any of the two approaches gives better results (VIS gives a slightly lower MAD and SIS gives a lower maximum error, but none of the differences is statistically significant) and there is no trend in the calculated affinities.

**Table 1:** Relative Binding Free Energies (kJ/mol) for the Four Ligands of BRD4, Showing the Results of Five Independent Simulations with Different Starting Velocities or Using Five Different Solvent Boxes^a^

	L1 → L3	L3 → L2	L3 → L4	L4 → L2
VIS				
1	1.45 ± 0.10	6.62 ± 0.17	–0.38 ± 0.08	9.34 ± 0.16
2	1.18 ± 0.10	6.62 ± 0.17	–0.10 ± 0.08	8.06 ± 0.17
3	1.80 ± 0.10	8.24 ± 0.16	0.00 ± 0.07	7.16 ± 0.17
4	1.30 ± 0.10	7.08 ± 0.17	0.09 ± 0.08	8.71 ± 0.17
5	1.40 ± 0.10	8.04 ± 0.16	0.03 ± 0.08	9.09 ± 0.17
Av	1.43 ± 0.10	7.32 ± 0.35	–0.07 ± 0.08	8.47 ± 0.39
Err	0.17	0.2	–0.07	1.78
Qual	0.7 ± 0.2	1.8 ± 0.7	1.00 ± 0.01	1.00 ± 0.02
SIS				
1	1.59 ± 0.10	9.01 ± 0.16	–0.30 ± 0.08	8.69 ± 0.17
2	1.66 ± 0.10	8.11 ± 0.16	–0.56 ± 0.08	8.49 ± 0.17
3	1.49 ± 0.10	7.24 ± 0.17	0.26 ± 0.08	8.59 ± 0.17
4	1.76 ± 0.10	6.47 ± 0.17	–0.11 ± 0.08	7.11 ± 0.17
5	1.32 ± 0.10	9.66 ± 0.16	–0.10 ± 0.08	7.62 ± 0.17
Av	1.65 ± 0.07	8.10 ± 0.58	**–**0.16 ± 0.13	8.10 ± 0.31
Err	0.31	1.41	–0.16	1.41
Qual	0.8 ± 0.3	1.4 ± 0.6	1.00 ± 0.01	1.00 ± 0.02

a Av shows the average results and standard error of these five independent simulations. Err is the error compared to the experimental affinities (1.26, 6.69, 0.00, and 6.69 kJ/mol for the four transformations).^38^ The four quality measures (Qual) are MAD, Max, *R*^2^, and τ_*r*90_, in this order.

Three of the transformations form a thermodynamic cycle, which should give a net vanishing free energy, L3 → L2 → L4 → L3. Forming all (125) possible sums of the five independent results of three transformations give results that vary from −2.8 to 1.5 kJ/mol with an average of −1.08 ± 0.5 for VIS and from −2.5 to 3.1 kJ/mol with an average of 0.16 ± 0.7 for SIS. The latter is clearly vanishing within the statistical uncertainty, whereas the former may have a slight bias (it differs from zero with 93% probability). This may indicate that the SIS approach gives a better ensemble of independent simulations.

The precision of the individual affinities was estimated by MBAR. It can be seen that it is rather even for all perturbations, 0.07–0.17 kJ/mol. The precision is very similar for VIS and SIS and vary much more between the transformations than between the two approaches. The precision of the net estimate of the binding affinity (the average) is calculated as the standard error over the five independent estimates (the standard deviation divided by ). It can be seen that it is typically somewhat larger than the precision estimated by MBAR (with only one exception), by a factor of 1.1–3.5. This shows that a single FEP calculation gives an overoptimistic estimate of the precision and that individual simulation may give ΔΔ*G* results that differ by 0.4–3.2 kJ/mol. Undoubtedly, several independent simulations are needed to obtain a true estimate of both the binding free energy and the precision of that estimate, as has been pointed out in several previous investigations.^33,34,36,37,39−41^

It can also be seen that VIS and SIS give rather similar estimates of the precision. Both approaches agree that the L3 → L2 and the L4 → L2 transformations, involving a change from a H atom to a six-membered ring, give the largest uncertainty. The estimated uncertainty is similar (within 0.1 kJ/mol) for the two approaches for three of the transformations, whereas for the L3 → L2 transformation, SIS gives an uncertainty (0.58 kJ/mol) that is almost two times larger than that obtained with VIS (0.35 kJ/mol).

For T4 lysozyme, we studied six transformations involving the seven ligands also shown in Figure 1. The results are collected in Table 2. It can be seen that the two approaches give statistically coinciding results for five of the transformations (i.e., the averages coincide within twice the uncertainty); the averages agree within 0.7 kJ/mol. However, for the Eth → Tol transformation, the results differ significantly, 2.5 ± 0.1 and 3.1 ± 0.1, giving a difference that is four times larger than the uncertainty, (0.61 ± 0.15 kJ/mol). The VIS calculations give free-energy differences of 2.4–2.9 kJ/mol, whereas those with the SIS calculations are 2.8–3.3 kJ/mol. Of course, the difference can be caused by random fluctuations caused by the rather small sample size, but a simple simulation with Gaussian random numbers shows that if the two distributions are identical (with an average of 2.82 and a standard deviation 0.39 kJ/mol), it is only 1.4% chance that such a large difference is observed between two averages of five samples. Therefore, it is more likely that the default solvent box in AMBER gives results that are significantly lower than for other random solvent boxes for this transformation. We have checked the conformation of the ethyl group in theses simulations, but as can be seen from the results in Table S2, there is no difference in the conformations between the two methods.

**Table 2:** Relative Binding Free Energies (kJ/mol) for the Seven Ligands of T4 Lysozyme, Showing the Results of Five Independent Simulations with Different Starting Velocities or Using Five Different Solvent Boxes^a^

	Ben → Phe	Eth → Tol	Tol → Ben	Ide → Ido	Ido → Bzf	Ide → Bzf
VIS						
1	8.07 ± 0.06	2.36 ± 0.08	–0.19 ± 0.07	7.63 ± 0.06	–11.92 ± 0.05	–3.78 ± 0.06
2	7.97 ± 0.07	2.50 ± 0.08	–0.20 ± 0.07	7.62 ± 0.06	–11.73 ± 0.05	–3.86 ± 0.06
3	8.42 ± 0.06	2.38 ± 0.08	–0.08 ± 0.07	6.61 ± 0.06	–11.51 ± 0.05	–3.99 ± 0.06
4	8.02 ± 0.07	2.41 ± 0.08	–0.16 ± 0.07	8.01 ± 0.06	–9.81 ± 0.05	–4.20 ± 0.06
5	8.07 ± 0.07	2.91 ± 0.08	–0.33 ± 0.07	7.75 ± 0.06	–11.48 ± 0.05	–3.87 ± 0.06
Av	8.11 ± 0.08	2.51 ± 0.10	**–**0.19 ± 0.04	7.52 ± 0.24	**–**11.29 ± 0.38	**–**3.94 ± 0.07
Err	–2.14	1.51	–1.57	6.52	–8.91	–2.56
Qual	3.9 ± 0.3	8.9 ± 0.4	0.50 ± 0.04	0.67 ± 0.06		
SIS						
1	8.02 ± 0.07	3.29 ± 0.08	–0.38 ± 0.07	7.29 ± 0.06	–12.09 ± 0.05	–4.42 ± 0.06
2	8.09 ± 0.07	2.84 ± 0.08	–0.51 ± 0.07	7.00 ± 0.07	–11.92 ± 0.05	–4.37 ± 0.07
3	8.04 ± 0.07	2.91 ± 0.08	–0.07 ± 0.07	7.67 ± 0.06	–11.59 ± 0.05	–3.62 ± 0.07
4	8.15 ± 0.07	3.26 ± 0.08	–0.72 ± 0.07	7.01 ± 0.06	–11.52 ± 0.05	–4.12 ± 0.06
5	8.42 ± 0.07	3.33 ± 0.08	0.39 ± 0.07	7.35 ± 0.06	–12.90 ± 0.05	–4.26 ± 0.06
Av	8.14 ± 0.07	3.13 ± 0.10	**–**0.26 ± 0.19	7.26 ± 0.12	**–**12.01 ± 0.25	**–**4.16 ± 0.14
Err	–2.11	2.12	–1.64	6.26	–9.62	–2.78
Qual	4.1 ± 0.3	9.6 ± 0.4	0.48 ± 0.04	1.00 ± 0.03		

a Av shows the average results and standard error of these five independent simulations. Err is the error compared to the experimental affinities (≥10.25, 1.00, 1.38, 1.00, – 2.38, and −1.38 kJ/mol for the six transformations).^46,72^ The four quality measures (Qual) are MAD, Max, *R*^2^, and τ_*r*90_, in this order.

Compared to the experimental estimates,^46^ the results are appreciably worse than for the BRD4 simulations. For four of the transformations, the errors are 1.5–2.8 kJ/mol (in absolute terms). However, for the Ide → Ido and Ido → Bzf transformations, the errors are high, 6–10 kJ/mol. Both transformations involve indole and the transformation of the heterocyclic NH group to either CH_2_ or O. Together with Ide → Bzf, these three transformations form a thermodynamic cycle, which should give a net vanishing free energy. Indeed, the two approaches give average cycle free energies of 0.17 ± 0.5 and −0.59 ± 0.3 kJ/mol, which are reasonably close to zero. This indicates that the sampling is reasonably converged (although a vanishing cycle free energy is only a sufficient criterion for convergence) and that the problem may instead be caused by the force field or by the starting poses of the molecules (or possibly by errors in the experimental estimates).

The quality of the predictions from the VIS and SIS approaches is similar. VIS gives a lower MAD (3.9 ± 0.3 vs 4.1 ± 0.3) and maximum error (8.9 ± 0.4 vs 9.6 ± 0.4; i.e., none of the differences are statistically significant), but SIS gives a better τ_*r*90_ (0.67 ± 0.06 vs 1.00 ± 0.03). However, the latter difference is only caused by the slightly larger uncertainty for SIS for the Tol → Ben transformation, 0.19 kJ/mol, making the results, −0.26 kJ/mol, not statistically significant different from zero and therefore this estimate, with an incorrect sign, is not included in τ_*r*90_, whereas it is significant and with an incorrect sign for VIS, −0.19 ± 0.04.

The precision of the individual affinities are similar or better than those of the BRD4 simulations and also more similar between the six transformations, 0.05–0.08 kJ/mol. This reflects that the transformations are more similar, always involving the modification or addition of a single heavy atom. There is no difference in the precision of the two approaches. As for BRD4, the uncertainty estimated by MBAR for the individual simulations is significantly smaller than that estimated from the standard error over the five independent estimates (again with a single exception), by a factor of 1.1–7.9. The results of the individual simulations give results that differ by 0.4–2.1 kJ/mol.

Several researchers have studied the binding of ligands to this lysozyme T4 mutant. They have observed that the side chain of one of the residues close to the binding site, Val-111, shows slow dynamics that may affect the binding affinity.^69−71^ We also observe slow sampling of the Val-111 side chain in our simulations, as is shown in Table S3. In most simulations, almost only the trans conformation is observed, with only 1–4% of the gauche^–^ conformation. However, occasionally, the amount of the latter conformation is higher (up to 19%) and in ∼20% of the simulations, the other gauche conformations is encountered or even dominates. We do not see any difference in the sampling between the VIS and SIS simulations (which was not expected since they differ only in the starting conformation of solvation-water molecules), nor do we see any correlation between the conformation of Val-111 and the relative binding affinity obtained, because the free energies are calculated from 11 simulations with different λ values and varying sampling of the Val-111 conformation.

We have also studied the ligand dynamics in the binding site by following six distances between ligand atoms in the benzene ring and protein atoms, approximately forming a box around the ligand. The results in Table S4 again do not indicate any significant differences between VIS and SIS. The small benzene and phenol ligands show the largest dynamics with standard deviations larger than 0.6 Å for five of the distances, whereas the standard deviation for most of the other simulations is 0.2–0.5 Å. However, occasionally larger fluctuations are observed also in some of the other transformations, but without any clear correlation to the calculated ΔΔ*G* or the type of independent simulations.

In general, the two approaches to generate independent simulations give similar estimates of the precision. The largest difference is for the Tol → Ben transformation, for which SIS gives 0.15 kJ/mol larger uncertainty than VIS.

To gain further statistics, we run VIS and SIS simulations on three additional proteins, viz. nine small substituted benzene ligands binding to a ferritin dimer (ten transformations),^50^ ten ligands binding to blood coagulation factor Xa (ten transformations),^49^ and 12 ligands binding to dihydrofolate reductase (15 transformations).^47,48^ The results for these calculations are shown in Tables S5–S7 and they are discussed in the Supporting Information. The results are completely analogous to those of BRD4 and lysozyme. VIS and SIS give similar results and a comparable precision. In one case (ferritin), there is an indication that SIS samples a slightly larger amount of the phase space.

## Conclusions

In this study, we have compared two different methods to generate independent simulations for the calculation of relative binding free energies, viz. using different starting velocity (VIS) or using different solvent boxes (SIS). We show that the two approaches in most cases give identical results. However, sometimes SIS gives a slightly larger variation and somewhat different results.

Our simulations clearly emphasize the importance of running independent simulations. Individual simulations can give relative binding free energies that differ by several kJ/mol, which is highly significant for such accurate FEP simulations and can affect the conclusions in drug-development projects.

It has previously been argued that a variation of starting velocities is enough to generate independent trajectories.^36,37,39−41^ Such a variation is easy to obtain by simply modifying a keyword in the input file. However, we would strongly argue that it is better to employ all random steps in the setup of the simulation to generate an ensemble of independent simulations with a maximum of possible variations. Molecular simulations are affected by both the random numbers employed in the setup and running of the simulations, as well as the parameters used for the simulations.^26^ The parameters can be both those connected to the method (simulation parameters) and those involved in the force field. An important arbitrary choice in the setup of a simulated system is the solvation of the solute. This is normally done by overlaying the solute with a periodic box of equilibrated water molecules. Of course, this is a crude and random approach for solvation–using different solvent boxes would give different results. We employed different solvent boxes, obtained from snapshots from an equilibrated MD simulation of pure water.^34^ Of course, this generates simulations with a varying number of water molecules, but this is natural because there is no simple method to determine the proper number of atoms in the solvated system. This means that there will be different topology and starting-coordinate files for each individual simulation, in variance to the velocity approach, which use the same topology and coordinates for the individual simulations. It should be pointed out that the SIS approach affects only the solvation water molecules, primarily outside the protein. If there are buried cavities in the protein that should be solvated during the simulation, enhanced-sampling methods need to be used, e.g., grand-canonical Monte Carlo simulations.^73−76^ However, the SIS approach may give a first indication if solvation of the protein is problematic for the calculated binding free energies (indicated by widely varying calculated energies).

There are also other steps of the protein setup that are random or at least very uncertain. If there are alternative conformations of groups in the macromolecule with equal occupancy, they are normally selected by random (or simply taking the first conformation for all). This is quite arbitrary and the selection could be used to enhance the sampling.^34^ However, groups of conformations are often connected, so a procedure that is more intelligent than a random choice would be desirable. The sampling can also enhanced by starting from different crystal structures.^77^

Another important choice is the positions of the hydrogen atoms (which are typically not seen in X-ray crystal structures). The positions of some of the protons can easily be deduced from the positions of the heavy atoms (e.g., aromatic, amide and CH_2_ groups). The positions of H atoms in CH_3_ groups are unknown, but probably not so important for calculated energies (and highly dynamic). On the other hand, the protons of alcohol and amine groups are important for hydrogen bonds and can in principle be placed anywhere on a circle around the heavy atom. Even worse, protons on water molecules can be placed anywhere on a sphere around the O atom and also form strong hydrogen bonds. There are more or less sophisticated algorithms in most MD and computational chemistry software to add such protons in proper positions. Likewise, these also try to determine the proper protonation state of all residue (in particular His residues) and sometimes they also consider the problem that crystallographic structures normally cannot differ between C, N and O atoms (affecting the side chains of His, Asn and Gln residues). These uncertainties differ from the starting velocities and solvation in that there exist solutions that are more correct or likely than other solutions and therefore the selection is not completely random. In a previous study, we employed Monte Carlo simulations to generate different starting protonation states, showing that they did not significantly affect binding affinities, except for residues close to the ligand-binding site.^34^ Another study has enhanced the sampling by using different protonation states of the catalytic Asp dyad in HIV-1 protease.^41^ The importance of software settings and force-field parameters have been studied for binding-energy calculations and MD simulations.^26,27^

Currently, we tend to recommend to employ as accurate algorithms as possible to protonate the starting structure and then employ velocities and different solvation boxes to generate independent simulations. However, with proper algorithms, it would be interesting to vary the positions of at least solvent-exposed alcoholic groups and water molecules in different simulations, as well as all residues with several equally likely conformations.

## Data Availability

The data supporting this article have been included in the Supporting Information.

## Supporting Information Available

Ligands, results and discussion of the FEP calculations for dihydrofolate reductase, factor Xa, and ferritin; variation of the ethyl dihedral angle in the Eth → Tol transformation of lysozyme; variation of the Val-111 dihedral angle in the lysozyme simulations; variation of the six key distances between the ligand and lysozyme in the simulations; AMBER parameters for the ligands (PDF)
