# Supplementary material for: Impact of Varying Velocities and Solvation Boxes on Alchemical Free-Energy Simulations
Source: J Chem Inf Model. 2025 Jan 31;65(4):2107–15. doi: 10.1021/acs.jcim.4c02236 (PMC11863368; doi:10.1021/acs.jcim.4c02236)
Supplement: Supplementary file 1 — ci4c02236_si_001.pdf [file ci4c02236_si_001.pdf]

Supplementary Information

Impact of Varying Velocities and Solvation Boxes on Alchemical Free Energy Simulations

Meiting Wang,[†,‡] Hao Jiang,[‡] and Ulf Ryde[*,‡]

[†]School of Medical Engineering & Xinxiang Key Laboratory of Biomedical Information Research & Henan International Joint Laboratory of Neural Information Analysis and Drug Intelligent Design & Xinxiang Key Laboratory of Biomedical Information Research, Xinxiang Medical University, Xinxiang 453003, China

[‡]Department of Computational Chemistry, Lund University, Chemical Centre, P.O. Box 124, SE-221 00 Lund, Sweden

E-mail: ulf.ryde@compchem.lu.se

Results for additional systems

To obtain further statistics, we repeated the same calculations for three more proteins, dihydrofolate reductase (DHFR), factor Xa and ferritin. The three test sets are taken from our previous study.[1, 2] The calculations were set up in a similar way as for BRD4 and lysozyme, except that 13 λ values were used and that the production run was 2 ns. The results are described in three separate sections. The ligands are shown in Figure S1 and are described in Table S1.

Results for DHFR

First, we studied 15 transformations for DHFR, involving 13 ligands of two different series (both with a net +1 charge). The results are collected in Table S2. It can be seen that the Δ*G* values obtained with the VIS and SIS approaches agree within 0.4 kJ/mol for 13 of the transformations. For the remaining two, 1c→1h and 2b→2e, the difference is 0.8 and 1.1 kJ/mol, but these differences are accompanied by larger uncertainties so that the differences are not statistically significant.

The calculated affinities mostly reproduce the experimental results within 5 kJ/mol. However, for two transformations, 2b→2e and 2c→2d, much larger errors are obtained, 19–20 and 12 kJ/mol and even the sign of Δ*G* is incorrect. Consequently, the correlation is poor, R^2 = 0.13–0.16, as is $\tau_{r,90}$ = 0.27. On the other hand, MAD is decent, 3.7–3.9 kJ/mol. SIS gives the larger error for the worst transformation and therefore also slightly poorer MAD and R^2, but none of the differences are statistically significant.

The transformations are designed so that they form five thermodynamic cycles. 1h→1f→1a→1c→1h and 1b→1a→1c→1b give small cycle Δ*G* values of 0.1–0.6 kJ/mol for both VIS and SIS. 2b→2a→2c→2b give slightly larger cycle Δ*G* energies of 0.8–1.0 kJ/mol, but they are still quite significant owing to the small uncertainties, 0.2–0.3 kJ/mol. For the 1h→1f→1e→1g→1h cycle, the cycle Δ*G* energy is even larger, 2.4–2.8±0.4 kJ/mol, which is strongly significant. Even worse, for the 2b→2c→2d→2e→2b cycle, the cycle free energy is very large 10.0±0.9 or 10.9±0.5 kJ/mol. The latter cycle includes the two transformations that gave very poor result. Apparently, the errors cancel, but only partly. However, it is clear that these transformations, involving the modification of two H atoms to methyl groups and possibly also a third H atom to a F atom, involve significant convergence problems, although the reason for this is currently unclear.

The MBAR uncertainty is identical for all VIS and SIS simulations and quite small, 0.03–0.3 kJ/mol. It is highest for the two problematic 2b→2e and 2c→2d transformations. The uncertainty estimated from the independent simulations is slightly larger (except for the 1a→1f transformation), 0.1–0.7 kJ/mol, again largest for the 2b→2e and 2c→2d transformations. There is no consistent difference in the uncertainty obtained with the VIS and SIS approaches.

Results for fXa

For fXa, we tested ten ligands in two series, one having a net charge of +1 (only two ligands) and the other having a net charge of +2. The results are shown in Table S3. It can be seen that the results of VIS and SIS are very similar. The estimated Δ*G* values agree within 0.6 kJ/mol for all transformations. The estimated affinities agree with the experimental ones within 4.4 kJ/mol for all transformations, except one (39→63), for which the error is 9.1–9.2 kJ/mol. Both methods give MADs of 2.3 kJ/mol and a perfect $\tau_{r,90}$, whereas SIS gives a slightly larger R^2 (0.29 compared to 0.27), but the maximum error is slightly larger.

We have designed the transformations for fXa so that they involve two thermodynamic cycles, 50→53→47→50 and 51→53→49→51. Both VIS and SIS give cycle Δ*G* values close to

zero, less than 0.4 kJ/mol (in absolute terms), with standard errors of the same size. Thus, the simulations seem to be properly converged.

The MBAR uncertainty of the individual transformations is 0.1–0.2 kJ/mol and equal for VIS and SIS. The uncertainty estimated from the independent simulations is similar (0.1–0.2 kJ/mol), except for four transformations, 39→63 for both VIS and SIS (0.4–0.5 kJ/mol), 53→51 for VIS and 53→49 for SIS (both 0.3 kJ/mol).

For one transformation (53→125), we tested to run individual SIS simulations with the same random seed for all five simulations. This gave essentially the same Δ*G* values as with different random seeds, within 0.4 kJ/mol. It should be noted that even if the same random seed gives the same starting velocities to all protein atoms and crystal-water molecules, the atom themselves are not at exactly the same positions, because our setup involves an initial restrained minimisation, which will give differing coordinates to all atoms since the solvation-water molecules are at different positions and the restraint force constant is 100 kcal/mol/Å^2. Therefore, we did not investigate further SIS simulations with the same random seed.

Results for ferritin

For ferritin, we examined ten transformations involving nine ligands, L1–L9. The results are shown in Table S4. It can be seen that the Δ*G* results vary somewhat more than for the other two proteins, both within the independent copies and between VIS and SIS. The precision estimated by MBAR is 0.1–0.2 kJ/mol for most of the transformations, but 0.3–0.4 kJ/mol for L5→L4 and L2→L4. These transformations involve the larger ligands, but L2→L3 transformation also involve large ligands, so it seems that the L4 ligand with one Pr and one i-Pr group gives rise to larger fluctuations than the other ligands.

For VIS, the L1→L7, L2→L4, L3→L1 and L4→L7 transformations give an uncertainty of 0.6–1.3 kJ/mol. The result for L2→L3 is intermediate (0.6 kJ/mol), whereas the other five transformations give a smaller uncertainty (0.3–0.4 kJ/mol). For SIS, only three transformations give a small uncertainty (L5→L6, L6→L8 and L9→L8; 0.2–0.3 kJ/mol), whereas that for the others is 0.7–1.5 kJ/mol. This indicates that SIS samples a slightly larger conformational space.

With five independent simulations, the average Δ*G* of VIS and SIS agree within 0.2–2.4 kJ/mol. The difference for the L5→L6 transformation is statistically significant at the 93% level, whereas the other differences are not significant. The estimated Δ*G* values agree with the experimental results within 0.3–7.6 kJ/mol. The largest error is for the L5→L4 transformation for both VIS and SIS They give a MAD of 2.3–2.7 kJ/mol, R^2 of 0.46–0.50 and a $\tau_{r,90}$ of 0.43. The former two are better for SIS, whereas the maximum error is smaller for VIS, but none of the differences is statistically significant.

The transformations are designed to form two thermodynamic cycles: L4→L7→L1→L3→L2→L4 and L8→L6→L5→L4→L7→L9→L8. The first cycle gives Δ*G* energies of 4.7±2.4 and 1.1±2.1 kJ/mol for VIS and SIS, respectively, which are not statistically significantly different from 0. However, for the second cycle, both VIS and SIS give statistically significant results, 6.4±1.3 and 5.8±1.7 kJ/mol, which indicates sampling issues, probably connected with the L5→L4 transformation that gave the largest error.

Thus, ferritin, gives larger differences between the individual simulations than the other systems and the sampling seems to be slightly larger for SIS than VIS.

Figure S1. Ligands used for DHFR, fXa and ferritin.

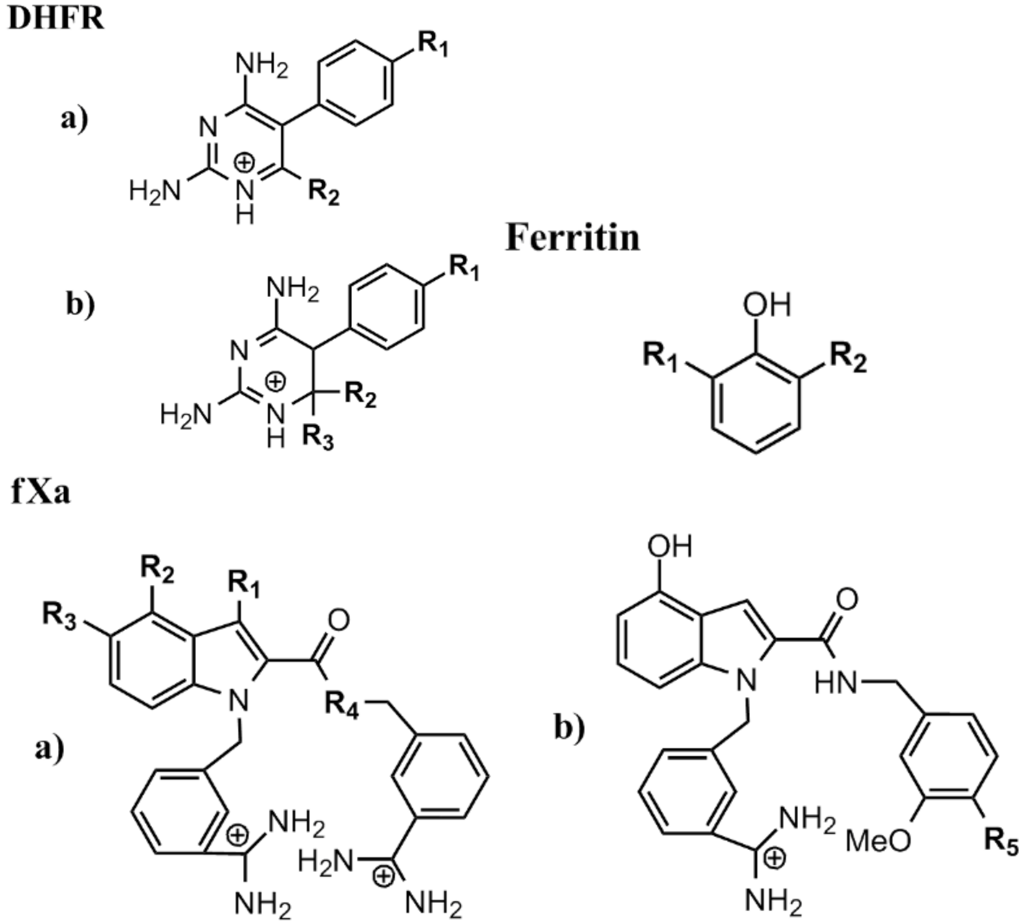

Table S1. Ligands studied, referring to Figure S1.

Protein	Ligand	Groups
DHFR[a]	1a	(Cl,Et)
	1b	(Br,Et)
	1c	(Me,Et)
	1e	(Cl,H)
	1f	(Cl,Me)
	1g	(H,H)
	1h	(H,Me)
	2a	(Cl,Me,Me)
	2b	(Me,Me,Me)
	2c	(H,Me,Me)
	2d	(H,H,H)
	2e	(F,H,H)
fXa	5	(Br,Me,H,NH)
	9	(Cl,H,H,NH)
	39[b]	OMe
	47	(H,Me,H,NH)
	49	(H,H,NH_2,NH)
	50	(H,OH,H,NH)
	51	(H,H,OH,NH)
	53	(H,H,H,NH)
	63[b]	H
	125	(H,H,H,O)
Ferritin	1	(i-Pr,i-Pr)
	2	(s-Bu,s-Bu)
	3	(s-Bu,i-Pr)
	4	(i-Pr,Pr)
	5	(Et,Et)
	6	(Et,Me)
	7	(i-Pr,H)
	8	(Me,Me)
	9	(H,H)

[a] Ligands starting with 1 and 2 are using the a) and b) structures of DHFR ligands in Figure S1, respectively.

[b] These two ligands use the b) structure of fXa in Figure 1, whereas the other fXa ligands use the a) structure

Table S2. Average (Av), standard deviation (Std), minimum (Min) and maximum (Max) of the C5–C6–C7–C8 dihedral angle in the Eth ligand (dihedral of the ethyl group) for the Eth→Tol lysozyme simulation. g^-, tr and g^+ show the percentage of the snapshots with the angle in the ranges –90 to 30º, 30–150º and 150–270º, respectively. Av in the main table is the average over the five VIS or SIS simulations and Diff if the difference between these two averages. Results are shown for 5000 snapshots from the λ = 0.0 simulations.

	Av	Stdev	Min	Max	g^-	tr	g^+
v1	88.5	30.5	-88.9	268.8	1.4	95.1	3.5
v2	89.4	31.9	-88.2	268.1	1.3	95.5	3.2
v3	88.2	36.0	-89.8	269.8	1.6	94.7	3.8
v4	88.5	35.0	-89.4	266.7	1.2	94.4	4.4
v5	89.4	39.5	-89.8	269.4	1.9	92.6	5.5
**Av**	**88.8**	**34.6**	**-89.2**	**268.6**	**1.5**	**94.5**	**4.1**
s1	88.2	19.5	-87.1	268.9	0.3	99.2	0.5
s2	88.5	25.9	-89.1	269.5	0.4	98.1	1.5
s3	87.9	31.4	-88.9	269.8	0.7	96.9	2.4
s4	88.4	28.3	-89.2	269.7	1.0	97.1	1.9
s5	86.8	26.0	-89.0	268.9	0.6	98.1	1.3
**Av**	**88.0**	**26.2**	**-88.7**	**269.4**	**0.6**	**97.9**	**1.5**
Diff	***-0.8***	***-8.4***	***0.6***	***0.8***	***-0.9***	***3.4***	***-2.5***

Table S3. Average (Av), standard deviation (Std), minimum (Min) and maximum (Max) of the HA–CA–CB–HB dihedral angle in Val-111 for the various lysozyme simulations. g^-, tr and g^+ are the percentage of snapshots with the angle in the ranges 0–120º, 120–240º and 240–360º, respectively. Av in the main table is the average over the five VIS or SIS simulations and Diff if the difference between these two averages. Results are shown for 5000 snapshots from the λ = 0.0 simulations.

	Av	Std	Min	Max	g^-	tr	g^+
Eth→Tol							
v1	171	16	56	212	2	98	0
v2	179	35	55	330	2	92	6
v3	171	17	44	219	2	98	0
v4	173	14	56	223	1	99	0
v5	173	16	49	221	2	98	0
**Av**	**174**	**19**	**52**	**241**	**2**	**97**	**1**
s1	173	15	44	215	1	99	0
s2	169	22	45	217	4	96	0
s3	172	14	40	213	1	99	0
s4	197	57	48	334	4	75	21
s5	174	10	95	207	0	100	0
**Av**	**177**	**24**	**55**	**237**	**2**	**94**	**4**
Diff	***3***	***4***	***3***	***-4***	***0***	***-3***	***3***
Ben→Phe							
v1	169	29	42	233	7	93	0
v2	171	26	35	212	5	95	0
v3	166	32	26	226	9	91	0
v4	174	21	32	222	3	97	0
**Av**	**170**	**27**	**34**	**223**	**6**	**94**	**0**
s1	154	44	30	235	19	81	0
s2	261	57	138	332	0	29	71
s3	175	12	124	222	0	100	0
s4	180	23	124	326	0	97	3
s5	180	12	133	237	0	100	0
**Av**	**190**	**29**	**110**	**270**	**4**	**81**	**15**
Diff	***20***	***3***	***76***	***47***	***-2***	***-13***	***15***
Tol→Ben							
v1	177	11	49	216	0	100	0
v2	172	16	41	222	1	99	0
v3	172	23	46	244	4	96	0
v4	171	35	40	332	7	91	3
v5	177	11	50	213	0	100	0
**Av**	**174**	**19**	**45**	**245**	**3**	**97**	**1**
s1	172	24	39	223	4	96	0
s2	176	12	57	215	1	99	0
s3	183	36	43	334	2	92	7
s4	173	21	37	217	3	97	0
s5	169	25	44	216	5	95	0

**Av**	**175**	**24**	**44**	**241**	**3**	**96**	**1**
Diff	***1***	***5***	***-1***	***-4***	***0***	***-1***	***1***
Ide→Ido							
v1	169	19	41	214	3	97	0
v2	218	65	46	333	2	62	37
v3	222	65	54	331	0	61	38
v4	172	12	51	207	0	100	0
v5	174	12	54	227	0	100	0
**Av**	**191**	**35**	**49**	**262**	**1**	**84**	**15**
s1	172	15	55	218	1	99	0
s2	167	21	47	220	4	96	0
s3	165	23	51	214	4	96	0
s4	170	19	45	208	2	98	0
s5	254	65	55	337	1	35	64
**Av**	**185**	**29**	**51**	**239**	**3**	**85**	**13**
Diff	***-6***	***-6***	***2***	***-23***	***1***	***1***	***-2***
Ido→Bzf							
v1	169	15	52	214	1	99	0
v2	183	37	116	335	0	92	8
v3	170	18	50	218	2	98	0
v4	172	14	39	211	1	99	0
v5	171	15	45	211	1	99	0
**Av**	**173**	**20**	**60**	**238**	**1**	**97**	**2**
s1	170	16	57	212	2	98	0
s2	172	12	47	210	1	100	0
s3	168	16	49	209	2	98	0
s4	168	19	53	215	3	97	0
s5	172	13	49	212	1	99	0
**Av**	**170**	**15**	**51**	**211**	**1**	**99**	**0**
Diff	***-3***	***-4***	***-9***	***-26***	***0***	***1***	***-2***
Ide→Bzf							
v1	170	19	40	217	2	98	0
v2	172	13	44	217	1	99	0
v3	170	17	49	238	2	98	0
v4	170	16	53	218	2	98	0
v5	164	25	46	211	6	94	0
**Av**	**169**	**18**	**46**	**220**	**2**	**98**	**0**
s1	164	29	47	216	8	92	0
s2	293	36	127	341	0	8	92
s3	240	65	43	335	0	49	51
s4	164	29	40	216	8	92	0
s5	171	18	48	216	2	98	0
**Av**	**206**	**35**	**61**	**265**	**4**	**68**	**29**
Diff	***37***	***17***	***14***	***45***	***1***	***-30***	***29***

Table S4. Average (Av), standard deviation (Std), minimum (Min) and maximum (Max) distance between the ligand atoms (C1–C6 in the main benzene ring, where C6 is connected to the sidechain) and six nearby atoms in lysozyme, approximately forming a box around the ligand. Av in the table is the average over the five VIS or SIS simulations and Diff if the difference between these two averages. Results are shown for 5000 snapshots from the λ = 0.0 simulation.

	C1–CB Ala98				C5–Cd2 Leu118				C1–CG1 Val111				C4–CG1 Val87				C3–N Tyr88				C6–SD Met102			
	Av	Std	Min	Max	Av	Std	Min	Max	Av	Std	Min	Max	Av	Std	Min	Max	Av	Std	Min	Max	Av	Std	Min	Max
Ben→Phe																								
v1	3.8	0.3	3.0	5.3	4.3	0.5	3.0	7.8	4.9	0.8	3.2	7.8	5.1	0.9	3.2	7.7	5.8	1.0	3.4	8.5	6.1	1.1	3.5	9.2
v2	3.8	0.3	3.0	5.1	4.2	0.5	2.9	6.1	4.7	0.8	3.2	7.2	5.1	1.0	3.2	7.8	5.6	1.0	3.5	8.4	6.0	1.1	3.5	9.0
v3	3.8	0.3	3.1	5.4	4.3	0.6	3.0	6.7	4.9	0.8	3.1	7.5	5.2	1.0	3.2	7.7	5.8	1.1	3.5	8.6	6.0	1.1	3.4	9.1
v4	3.8	0.3	3.0	5.3	4.3	0.6	3.0	6.5	4.8	0.8	3.2	7.7	5.1	1.0	3.2	8.1	5.6	1.0	3.3	8.8	6.0	1.1	3.4	9.0
**Av**	**3.8**	**0.3**	**3.0**	**5.3**	**4.3**	**0.6**	**3.0**	**6.8**	**4.8**	**0.8**	**3.2**	**7.6**	**5.1**	**1.0**	**3.2**	**7.8**	**5.7**	**1.0**	**3.4**	**8.6**	**6.0**	**1.1**	**3.5**	**9.1**
s1	3.8	0.3	3.1	5.5	4.2	0.5	3.1	6.7	4.8	0.8	3.2	7.1	5.1	1.0	3.3	7.6	5.6	1.0	3.4	8.2	5.9	1.1	3.5	8.9
s2	3.8	0.3	3.1	5.4	4.3	0.5	2.9	6.9	4.8	0.8	3.2	7.1	5.1	0.9	3.2	7.8	5.8	1.0	3.5	8.3	6.1	1.1	3.6	9.2
s3	3.8	0.3	3.1	5.1	4.2	0.5	3.0	6.1	4.7	0.8	3.1	7.0	5.0	1.0	3.2	7.2	5.5	1.0	3.4	7.9	5.9	1.1	3.4	8.9
s4	3.8	0.3	3.1	7.1	4.3	0.6	3.0	6.9	4.8	0.9	3.0	7.4	5.1	1.0	3.2	7.4	5.7	1.0	3.4	9.6	5.9	1.1	3.4	9.4
s5	3.8	0.3	3.0	5.0	4.3	0.6	3.1	6.9	4.8	0.8	3.2	6.7	5.2	1.0	3.3	7.6	5.6	1.0	3.5	9.0	6.0	1.1	3.4	9.1
**Av**	**3.8**	**0.3**	**3.1**	**5.6**	**4.3**	**0.5**	**3.0**	**6.7**	**4.8**	**0.8**	**3.1**	**7.1**	**5.1**	**1.0**	**3.2**	**7.5**	**5.6**	**1.0**	**3.4**	**8.6**	**6.0**	**1.1**	**3.5**	**9.1**
Diff	***0.0***	***0.0***	***-0.1***	***-0.3***	***0.0***	***0.0***	***0.0***	***0.1***	***0.0***	***0.0***	***0.0***	***0.5***	***0.0***	***0.0***	***0.0***	***0.3***	***0.1***	***0.0***	***0.0***	***0.0***	***0.1***	***0.0***	***0.0***	***0.0***
Eth→Tol																								
v1	3.6	0.2	3.0	4.9	3.7	0.3	2.9	5.9	3.7	0.3	3.0	5.6	4.0	0.3	3.2	5.6	4.7	0.4	3.2	6.9	5.2	0.4	4.1	7.2
v2	3.6	0.2	3.0	4.9	3.6	0.3	3.0	5.7	3.7	0.3	3.0	6.0	4.0	0.3	3.2	5.4	4.7	0.4	3.4	6.5	5.2	0.3	4.1	7.5
v3	3.6	0.2	3.1	4.7	3.6	0.3	3.0	5.4	3.7	0.2	3.0	5.3	4.0	0.3	3.2	5.4	4.7	0.4	3.5	6.2	5.2	0.3	4.2	6.6
v4	3.6	0.2	3.0	4.8	3.6	0.3	3.0	5.4	3.6	0.2	3.0	4.7	4.0	0.3	3.2	6.2	4.7	0.4	3.5	6.9	5.2	0.3	4.1	6.8
v5	3.6	0.2	3.0	5.5	3.7	0.3	3.0	5.7	3.6	0.2	3.0	5.5	4.0	0.3	3.2	5.8	4.7	0.4	3.3	6.5	5.2	0.4	4.1	6.8
**Av**	**3.6**	**0.2**	**3.0**	**5.0**	**3.6**	**0.3**	**3.0**	**5.6**	**3.7**	**0.2**	**3.0**	**5.4**	**4.0**	**0.3**	**3.2**	**5.7**	**4.7**	**0.4**	**3.4**	**6.6**	**5.2**	**0.3**	**4.1**	**7.0**
s1	3.6	0.2	3.0	5.1	3.7	0.3	3.0	5.6	3.7	0.2	3.1	5.3	4.0	0.3	3.2	5.6	4.6	0.4	3.4	5.9	5.1	0.3	4.2	6.9
s2	3.7	0.2	3.0	5.0	3.7	0.3	2.9	5.9	3.8	0.3	3.1	5.9	3.9	0.3	3.2	5.1	4.6	0.4	3.3	6.3	5.2	0.3	4.0	6.7
s3	3.6	0.2	3.0	4.6	3.7	0.3	3.0	5.7	3.7	0.2	3.0	5.2	4.0	0.3	3.3	5.5	4.6	0.4	3.3	6.0	5.2	0.3	4.1	7.0
s4	3.7	0.2	3.0	5.1	3.7	0.3	3.0	5.4	3.9	0.5	3.0	6.6	3.9	0.3	3.2	5.3	4.7	0.4	3.5	6.7	5.2	0.3	4.3	6.7
s5	3.6	0.2	3.0	4.8	3.9	0.7	3.0	7.6	3.7	0.2	3.1	4.7	4.0	0.3	3.2	5.3	4.7	0.4	3.4	6.3	5.1	0.3	4.1	6.7
**Av**	**3.6**	**0.2**	**3.0**	**4.9**	**3.7**	**0.4**	**3.0**	**6.0**	**3.8**	**0.3**	**3.1**	**5.5**	**4.0**	**0.3**	**3.2**	**5.4**	**4.6**	**0.4**	**3.4**	**6.2**	**5.2**	**0.3**	**4.1**	**6.8**
Diff	***0.0***	***0.0***	***0.0***	***0.0***	***0.1***	***0.1***	***0.0***	***0.4***	***0.1***	***0.0***	***0.1***	***0.1***	***0.0***	***0.0***	***0.0***	***-0.3***	***-0.1***	***0.0***	***0.0***	***-0.4***	***0.0***	***0.0***	***0.0***	***-0.2***

Tol→Ben																								
v1	3.6	0.2	3.0	4.4	3.7	0.3	2.9	5.7	3.8	0.2	3.0	5.7	4.0	0.3	3.2	6.0	4.6	0.4	3.5	6.7	5.1	0.3	4.1	7.1
v2	3.8	0.3	3.0	6.5	4.5	0.7	3.1	7.7	5.1	0.9	3.0	7.5	5.9	1.2	3.3	8.0	6.8	1.3	3.5	9.5	6.2	0.7	4.1	8.5
v3	3.8	0.4	2.9	6.0	4.3	0.8	2.9	8.1	4.2	0.8	3.0	6.8	5.0	1.4	3.1	8.4	5.8	1.6	3.4	9.8	5.7	0.9	4.0	8.5
v4	3.9	0.4	3.1	6.2	4.4	0.6	3.0	8.2	4.9	0.9	3.0	8.2	6.0	1.3	3.2	8.2	7.0	1.5	3.5	9.7	6.3	0.7	4.1	8.6
v5	3.6	0.2	3.0	4.5	3.7	0.3	2.9	5.6	3.7	0.2	3.0	5.2	4.0	0.3	3.1	6.5	4.6	0.4	3.5	6.8	5.2	0.4	4.0	7.4
**Av**	**3.7**	**0.3**	**3.0**	**5.5**	**4.1**	**0.5**	**3.0**	**7.1**	**4.3**	**0.6**	**3.0**	**6.7**	**5.0**	**0.9**	**3.2**	**7.4**	**5.8**	**1.0**	**3.5**	**8.5**	**5.7**	**0.6**	**4.1**	**8.0**
s1	3.6	0.2	3.0	5.2	3.7	0.3	3.0	6.2	3.7	0.2	3.0	5.8	4.0	0.3	3.2	6.5	4.6	0.4	3.5	7.9	5.2	0.4	4.0	6.9
s2	3.6	0.2	3.0	5.0	3.7	0.4	2.9	5.8	3.8	0.5	3.0	6.5	4.1	0.6	3.2	7.6	4.7	0.7	3.5	8.6	5.2	0.5	4.0	7.8
s3	3.6	0.3	3.0	6.1	3.8	0.5	3.0	6.9	3.8	0.4	3.0	6.6	4.0	0.3	3.1	6.4	4.7	0.4	3.3	7.0	5.2	0.3	4.0	6.8
s4	3.6	0.2	3.1	4.9	3.6	0.3	3.0	5.5	3.7	0.2	3.1	5.3	4.0	0.3	3.1	5.6	4.6	0.4	3.5	6.6	5.1	0.4	4.1	8.0
s5	3.7	0.2	3.0	4.9	3.8	0.4	3.0	5.9	3.9	0.5	3.1	6.9	4.1	0.8	3.1	8.1	4.8	0.9	3.4	8.7	5.3	0.5	4.1	7.3
**Av**	**3.6**	**0.2**	**3.0**	**5.2**	**3.7**	**0.4**	**3.0**	**6.1**	**3.8**	**0.4**	**3.0**	**6.2**	**4.0**	**0.5**	**3.1**	**6.8**	**4.7**	**0.6**	**3.4**	**7.8**	**5.2**	**0.4**	**4.0**	**7.4**
Diff	***0.1***	***0.1***	***0.0***	***0.3***	***0.4***	***0.2***	***0.0***	***1.0***	***0.6***	***0.2***	***0.0***	***0.5***	***0.9***	***0.4***	***0.0***	***0.6***	***1.1***	***0.5***	***0.0***	***0.7***	***0.5***	***0.2***	***0.0***	***0.7***
Ide→Ido																								
v1	4.2	0.3	3.3	5.3	3.8	0.3	3.2	5.4	5.7	0.4	4.6	7.8	6.2	0.3	4.9	7.6	6.2	0.3	5.0	7.8	7.4	0.3	6.3	8.5
v2	4.1	0.3	3.2	5.5	3.8	0.3	3.0	5.2	6.5	1.1	4.6	9.7	6.1	0.3	5.0	7.5	6.1	0.4	4.1	8.4	7.2	0.4	4.7	8.4
v3	4.2	0.3	3.3	5.7	3.9	0.4	3.2	7.0	6.5	1.0	4.5	9.3	6.2	0.3	4.4	7.5	6.2	0.4	4.0	8.4	7.3	0.4	4.3	9.1
v4	4.4	0.4	3.3	7.3	4.0	0.6	3.1	7.4	5.5	0.5	3.5	7.5	6.0	0.4	4.3	7.3	6.2	0.4	4.4	8.5	7.3	0.3	5.2	8.6
v5	4.1	0.5	3.2	6.8	4.7	1.0	3.1	8.0	5.4	0.6	3.1	7.7	5.6	0.7	3.6	7.4	6.1	0.5	3.5	8.1	6.6	1.0	3.5	8.6
**Av**	**4.2**	**0.4**	**3.3**	**6.1**	**4.0**	**0.5**	**3.1**	**6.6**	**5.9**	**0.7**	**4.1**	**8.4**	**6.0**	**0.4**	**4.4**	**7.5**	**6.2**	**0.4**	**4.2**	**8.2**	**7.2**	**0.5**	**4.8**	**8.6**
s1	4.2	0.3	3.2	5.5	3.9	0.3	3.1	5.8	5.7	0.6	3.1	8.0	6.0	0.5	3.6	7.3	6.2	0.4	4.7	8.3	7.2	0.7	3.5	9.0
s2	4.2	0.3	3.3	5.4	3.9	0.3	3.2	5.8	5.8	0.4	4.1	7.9	6.1	0.3	4.9	7.2	6.2	0.4	4.2	8.2	7.3	0.3	5.0	8.9
s3	4.2	0.3	3.2	7.4	4.0	0.4	3.1	6.7	5.5	0.7	3.1	7.7	6.0	0.6	3.6	7.6	6.3	0.5	3.8	8.8	7.0	0.9	3.6	9.6
s4	4.3	0.3	3.2	5.6	3.9	0.3	3.1	5.3	5.8	0.4	4.2	8.5	6.2	0.3	4.8	7.4	6.2	0.4	4.2	7.7	7.3	0.3	5.1	8.7
s5	4.3	0.3	3.1	5.6	3.8	0.3	3.1	5.0	6.9	1.0	4.4	9.1	6.1	0.3	4.6	7.5	6.2	0.5	3.7	8.6	7.2	0.4	5.1	9.0
**Av**	**4.2**	**0.3**	**3.2**	**5.9**	**3.9**	**0.3**	**3.1**	**5.7**	**5.9**	**0.6**	**3.8**	**8.2**	**6.1**	**0.4**	**4.3**	**7.4**	**6.2**	**0.4**	**4.1**	**8.3**	**7.2**	**0.5**	**4.5**	**9.0**
Diff	***0.0***	***0.1***	***0.1***	***0.2***	***0.1***	***0.2***	***0.0***	***0.9***	***0.0***	***0.1***	***0.3***	***0.2***	***-0.1***	***0.0***	***0.1***	***0.1***	***-0.1***	***0.0***	***0.1***	***-0.1***	***0.0***	***0.0***	***0.3***	***-0.4***
Ido→Bzf																								
v1	4.2	0.3	3.3	5.3	3.7	0.3	3.0	5.2	5.9	0.4	4.4	8.3	6.1	0.3	5.0	7.5	6.3	0.3	4.8	7.7	7.2	0.3	6.3	9.5
v2	4.1	0.3	3.2	5.3	3.7	0.3	3.0	5.3	6.0	0.6	4.3	8.8	6.1	0.3	5.2	7.3	6.2	0.3	4.7	7.3	7.2	0.3	6.2	8.5
v3	4.1	0.3	3.3	5.2	3.7	0.3	3.0	5.1	5.9	0.4	4.1	8.1	6.1	0.3	5.1	7.2	6.2	0.3	4.9	7.4	7.2	0.2	5.9	8.4
v4	4.8	0.5	3.4	6.9	5.0	1.0	3.0	7.8	4.8	0.7	3.6	7.8	5.6	0.6	3.8	7.1	6.5	0.5	4.9	8.3	7.0	0.5	5.3	9.3
v5	4.1	0.3	3.2	5.3	3.7	0.3	3.0	5.9	5.8	0.4	4.4	8.0	6.1	0.3	5.2	7.3	6.1	0.3	5.2	7.3	7.2	0.3	6.3	9.0
**Av**	**4.3**	**0.3**	**3.3**	**5.6**	**4.0**	**0.4**	**3.0**	**5.9**	**5.7**	**0.5**	**4.2**	**8.2**	**6.0**	**0.4**	**4.9**	**7.3**	**6.3**	**0.3**	**4.9**	**7.6**	**7.2**	**0.3**	**6.0**	**8.9**
s1	4.2	0.3	3.2	5.5	3.7	0.3	3.0	5.8	5.8	0.4	4.2	8.1	6.1	0.3	4.6	7.4	6.2	0.3	5.0	7.6	7.2	0.3	6.4	9.2

s2	4.1	0.3	3.2	5.3	3.7	0.3	2.9	5.3	5.9	0.4	4.7	7.9	6.1	0.3	4.8	7.4	6.2	0.3	5.0	7.9	7.2	0.3	6.3	8.5
s3	4.2	0.3	3.3	5.4	3.8	0.3	3.1	5.9	5.8	0.4	4.0	8.3	6.1	0.3	4.9	7.8	6.3	0.4	4.6	8.0	7.2	0.3	4.5	8.9
s4	4.2	0.3	3.4	5.6	3.7	0.3	3.1	7.1	5.8	0.4	4.6	8.4	6.1	0.3	4.9	7.1	6.2	0.3	5.0	7.5	7.2	0.3	6.5	8.6
s5	4.1	0.3	3.3	5.3	3.8	0.3	3.0	5.8	5.8	0.4	4.6	7.6	6.1	0.3	5.1	7.1	6.2	0.3	5.2	7.4	7.2	0.3	6.4	8.5
**Av**	**4.2**	**0.3**	**3.3**	**5.4**	**3.7**	**0.3**	**3.0**	**6.0**	**5.8**	**0.4**	**4.4**	**8.1**	**6.1**	**0.3**	**4.9**	**7.4**	**6.2**	**0.3**	**5.0**	**7.7**	**7.2**	**0.3**	**6.0**	**8.7**
Diff	***0.1***	***0.0***	***0.0***	***0.2***	***0.2***	***0.1***	***0.0***	***-0.1***	***-0.1***	***0.1***	***-0.3***	***0.1***	***-0.1***	***0.1***	***0.0***	***-0.1***	***0.0***	***0.0***	***-0.1***	***-0.1***	***0.0***	***0.0***	***0.0***	***0.2***
Ide→Bzf																								
v1	4.3	0.3	3.3	6.2	4.0	0.6	3.1	7.3	5.8	0.4	3.8	8.2	6.2	0.3	4.9	7.3	6.2	0.3	5.0	7.8	7.3	0.3	5.7	8.9
v2	4.4	0.4	3.2	6.0	3.9	0.3	3.2	5.2	5.7	0.4	4.4	7.4	6.3	0.3	5.0	7.4	6.2	0.3	4.9	8.0	7.3	0.3	5.9	9.4
v3	4.3	0.3	3.4	5.7	3.9	0.3	3.1	5.7	5.7	0.4	4.5	7.8	6.2	0.3	4.4	7.9	6.2	0.3	4.3	8.0	7.3	0.3	5.4	9.5
v4	4.3	0.3	3.2	5.3	3.9	0.3	3.2	5.7	5.8	0.4	4.4	8.0	6.1	0.3	4.6	7.5	6.2	0.3	4.6	7.5	7.3	0.3	5.8	9.4
v5	4.2	0.3	3.2	6.0	4.0	0.5	3.1	7.1	5.7	0.5	3.6	7.9	6.1	0.4	4.1	7.4	6.3	0.4	4.3	7.8	7.3	0.3	5.2	8.8
**Av**	**4.3**	**0.3**	**3.3**	**5.8**	**3.9**	**0.4**	**3.1**	**6.2**	**5.7**	**0.4**	**4.1**	**7.9**	**6.2**	**0.3**	**4.6**	**7.5**	**6.2**	**0.3**	**4.6**	**7.8**	**7.3**	**0.3**	**5.6**	**9.2**
s1	4.2	0.3	3.3	5.4	3.9	0.3	3.1	5.3	5.8	0.4	4.1	8.7	6.1	0.3	4.8	7.2	6.3	0.4	4.9	8.3	7.3	0.3	5.8	8.9
s2	4.3	0.4	3.2	6.1	4.1	0.4	3.1	5.7	5.6	1.5	3.3	9.3	5.2	0.9	3.5	7.7	6.3	0.6	3.9	8.2	5.8	1.2	3.6	8.9
s3	4.1	0.4	3.2	7.0	5.1	1.5	3.2	9.1	6.2	1.4	3.1	9.1	5.7	0.6	3.4	7.7	6.3	0.6	3.8	9.6	6.4	1.0	3.4	8.9
s4	4.4	0.3	3.4	5.7	4.0	0.3	3.2	5.8	5.7	0.4	4.2	7.8	6.3	0.5	4.8	9.4	6.2	0.4	4.6	8.0	7.3	0.3	6.2	8.5
s5	4.4	0.4	3.3	6.0	3.9	0.3	3.2	5.3	5.9	0.4	4.4	8.1	6.2	0.3	4.9	7.8	6.2	0.4	3.5	8.2	7.3	0.3	5.0	9.2
**Av**	**4.3**	**0.4**	**3.3**	**6.0**	**4.2**	**0.6**	**3.2**	**6.2**	**5.8**	**0.8**	**3.8**	**8.6**	**5.9**	**0.5**	**4.3**	**8.0**	**6.3**	**0.5**	**4.1**	**8.5**	**6.8**	**0.6**	**4.8**	**8.9**
Diff	***0.0***	***0.0***	***0.0***	***-0.2***	***-0.3***	***-0.2***	***0.0***	***0.0***	***-0.1***	***-0.4***	***0.3***	***-0.7***	***0.3***	***-0.2***	***0.3***	***-0.5***	***0.0***	***-0.2***	***0.5***	***-0.6***	***0.5***	***-0.3***	***0.8***	***0.3***

Table S5. Relative binding free energies (kJ/mol) for the twelve ligands of DHFR, showing the results of five independent simulations with different starting velocities or using five different solvent boxes. Av shows the average results and standard error of these five independent simulations. Err is the error compared to the experimental affinities (3.0±0.9, 1.7±0.9, 1.0±0.9, –0.7±0.1, 8.2±0.4, 0.5±0.4, –7.1±0.4, –3.4±0.4, –4.2±0.4, –0.5±0.6, 6.0±0.7, 12.5±0.6, –6.5±0.8, 7.0±0.7 and 0.5±0.3 kJ/mol for the 15 transformations).[3, 4] The four quality measures (Qual) are MAD, Max (both in kJ/mol), R^2 and τ_{r90} in this order.

	1a→1f	1b→1a	1c→1a	1c→1b	1c→1h	1f→1e	1g→1e	1g→1h	1h→1f	2b→2a	2b→2c	2b→2e	2c→2a	2c→2d	2e→2d
VIS															
1	2.9±0.2	-0.6±0.03	-0.2±0.1	0.2±0.1	12.7±0.2	2.4±0.1	-10.7±0.1	-0.5±0.1	-9.4±0.1	-1.0±0.1	4.7±0.1	-4.2±0.3	-7.4±0.1	-5.8±0.2	2.5±0.1
2	2.5±0.2	-0.2±0.03	0.0±0.1	0.4±0.1	10.9±0.2	1.9±0.1	-10.6±0.1	-0.4±0.1	-9.3±0.1	-1.4±0.1	5.4±0.1	-6.4±0.3	-6.9±0.1	-5.6±0.2	2.5±0.1
3	2.7±0.2	-0.9±0.03	0.0±0.1	0.4±0.1	10.7±0.2	0.9±0.1	-10.9±0.1	-1.3±0.1	-9.6±0.1	-1.2±0.1	5.5±0.1	-7.1±0.3	-7.7±0.1	-4.2±0.2	2.2±0.1
4	2.5±0.2	-0.5±0.03	-0.1±0.1	0.3±0.1	12.0±0.2	1.3±0.1	-10.8±0.1	-0.2±0.1	-9.4±0.1	-1.1±0.1	5.4±0.1	-5.7±0.3	-7.5±0.1	-5.4±0.2	1.8±0.1
5	2.8±0.2	-0.5±0.03	0.1±0.1	0.6±0.1	11.4±0.2	2.6±0.1	-11.0±0.1	-0.1±0.1	-9.0±0.1	-1.2±0.1	5.8±0.1	-8.7±0.3	-7.2±0.1	-3.6±0.2	2.2±0.1
Av	2.7±0.1	-0.5±0.11	0.0±0.1	0.4±0.1	11.5±0.4	1.8±0.3	-10.8±0.1	-0.5±0.2	-9.3±0.1	-1.2±0.1	5.3±0.2	-6.4±0.7	-7.3±0.1	-4.9±0.4	2.2±0.1
Err	-0.3	-2.2	-1.0	1.1	3.3	1.4	-3.7	2.9	-5.1	-0.7	-0.7	-18.9	-0.9	-11.9	1.8
Qual	3.7±0.2	18.9±1.0	0.16±0.03	0.27±0.04											
SIS															
1	2.1±0.2	-0.6±0.03	0.1±0.1	1.0±0.1	11.9±0.2	0.9±0.1	-10.5±0.1	-0.6±0.1	-9.7±0.1	-1.3±0.1	5.1±0.1	-7.2±0.3	-6.5±0.1	-4.9±0.2	2.3±0.1
2	2.5±0.2	-0.5±0.03	0.0±0.1	0.3±0.1	13.0±0.2	2.6±0.1	-11.0±0.1	-1.2±0.1	-9.3±0.1	-1.1±0.1	5.0±0.1	-8.4±0.3	-8.1±0.1	-5.6±0.2	1.9±0.1
3	2.4±0.2	-0.5±0.03	0.1±0.1	0.1±0.1	12.6±0.2	1.5±0.1	-11.1±0.1	-0.8±0.1	-9.8±0.1	-1.0±0.1	5.9±0.1	-7.6±0.3	-8.1±0.1	-4.5±0.2	2.2±0.1
4	2.3±0.2	-0.6±0.03	-0.1±0.1	0.3±0.1	12.3±0.2	1.6±0.1	-10.4±0.1	-0.8±0.1	-9.3±0.1	-1.3±0.1	5.1±0.1	-8.2±0.3	-7.3±0.1	-5.4±0.2	2.8±0.1
5	2.0±0.2	-0.6±0.03	-0.3±0.1	0.1±0.1	11.9±0.2	1.7±0.1	-11.2±0.1	0.3±0.1	-9.3±0.1	-1.0±0.1	5.5±0.1	-6.4±0.3	-7.3±0.1	-6.1±0.2	2.2±0.1
Av	2.3±0.1	-0.6±0.03	0.0±0.1	0.4±0.2	12.3±0.2	1.6±0.3	-10.9±0.2	-0.6±0.3	-9.5±0.1	-1.2±0.1	5.3±0.2	-7.5±0.4	-7.5±0.3	-5.3±0.3	2.3±0.1
Err	-0.7	-2.3	-1.1	1.1	4.1	1.2	-3.7	2.7	-5.3	-0.7	-0.7	-20.0	-1.0	-12.3	1.8
Qual	3.9±0.1	20.0±0.7	0.13±0.02	0.27±0.04											

Table S6. Relative binding free energies (kJ/mol) for the ten ligands of fXa, showing the results of five independent simulations with different starting velocities or using five different solvent boxes. The last column shows the results of the 53→125 transformation with the same random seed for all calculations. Av shows the average results and standard error of these five independent simulations. Err is the error compared to the experimental affinities (–10.1, 4.9, 0.6, 1.0, –1.9, –2.5, 2.5, –1.9, 3.5, 1.0 and 1.0 kJ/mol for the ten transformations).[5] The four quality measures (Qual) are MAD, Max (both in kJ/mol), R^2 and τ_{r90} in this order.

	39→63	47→5	47→50	49→51	53→9	53→47	53→49	53→50	53→51	53→125	Same seed
VIS											
1	-0.4±0.2	0.9±0.2	2.1±0.2	1.6±0.2	-1.7±0.1	-2.0±0.1	0.0±0.1	0.5±0.1	0.7±0.2	0.6±0.1	0.8±0.1
2	-0.6±0.2	0.6±0.2	2.1±0.2	0.7±0.2	-1.9±0.1	-1.8±0.1	-0.7±0.1	0.4±0.1	0.8±0.2	0.5±0.1	
3	-1.3±0.2	0.7±0.2	1.9±0.2	0.8±0.2	-1.7±0.1	-2.1±0.1	0.1±0.1	-0.1±0.1	1.5±0.2	0.9±0.1	
4	0.5±0.2	0.2±0.2	1.6±0.2	0.6±0.2	-1.5±0.1	-1.9±0.1	-0.3±0.1	0.1±0.1	2.3±0.2	0.9±0.1	
5	-2.2±0.2	0.8±0.2	1.6±0.2	1.6±0.2	-1.7±0.1	-2.1±0.1	0.1±0.1	-0.2±0.1	0.8±0.2	0.8±0.1	
**Av**	**-0.8±0.5**	**0.6±0.1**	**1.9±0.1**	**1.1±0.2**	**-1.7±0.1**	**-2.0±0.1**	**-0.1±0.2**	**0.1±0.1**	**1.2±0.3**	**0.7±0.1**	
Err	9.2	-4.3	1.2	0.0	0.2	0.5	-2.6	2.0	-2.3	-0.2	
Qual	2.3±0.4	9.3±1.7	0.29±0.12	1.00±0.17							
SIS											
1	-2.5±0.2	0.1±0.2	1.6±0.2	1.4±0.2	-1.5±0.1	-2.2±0.1	-0.5±0.1	-0.5±0.1	1.4±0.2	0.9±0.1	0.8±0.1
2	-0.4±0.2	0.6±0.2	1.9±0.2	1.3±0.2	-2.0±0.1	-1.9±0.1	-0.9±0.1	-0.6±0.1	1.0±0.2	0.7±0.1	0.6±0.1
3	-0.8±0.2	0.4±0.2	2.1±0.2	1.1±0.2	-1.6±0.1	-2.0±0.1	-1.3±0.1	0.3±0.1	1.6±0.2	0.7±0.1	0.7±0.1
4	-0.2±0.2	0.7±0.2	1.3±0.2	2.5±0.2	-1.6±0.1	-1.8±0.1	-0.6±0.1	-0.3±0.1	0.8±0.2	0.7±0.1	0.9±0.1
5	-1.0±0.2	0.5±0.2	2.1±0.2	1.1±0.2	-1.6±0.1	-2.0±0.1	-0.5±0.1	0.4±0.1	0.7±0.2	1.0±0.1	0.6±0.1
**Av**	**-1.0±0.4**	**0.5±0.1**	**1.8±0.2**	**1.5±0.3**	**-1.7±0.1**	**-2.0±0.1**	**-0.8±0.2**	**-0.1±0.2**	**1.1±0.2**	**0.8±0.1**	**0.7±0.1**
Err	9.1	-4.4	1.2	0.5	0.2	0.5	-3.2	1.7	-2.4	-0.2	-0.7
Qual	2.3±0.4	9.1±1.7	0.27±0.11	1.00±0.13							

Table S7. Relative binding free energies (kJ/mol) for the nine ligands of ferritin, showing the results of five independent simulations with different starting velocities or using five different solvent boxes. Av shows the average results and standard error of these five independent simulations. Err is the error compared to the experimental affinities (3.1±0.2, –2.6±0.6, –0.4±0.5, 2.2±0.4, 3.1±0.2, –2.4±0.2, 2.2±0.2, 3.1±0.1, 8.8±0.1 and 4.1±0.1 kJ/mol for the ten transformations).[6] The four quality measures (Qual) are MAD, Max (both in kJ/mol), R^2 and τ_{r90} in this order.

	L1→L7	L2→L3	L2→L4	L3→L1	L4→L7	L5→L4	L5→L6	L6→L8	L7→L9	L9→L8
VIS										
1	0.6±0.2	-0.1±0.2	-2.3±0.4	-0.5±0.2	-1.9±0.2	5.8±0.3	1.2±0.1	1.8±0.1	11.9±0.2	-5.0±0.2
2	-0.5±0.2	-3.0±0.2	-4.2±0.4	0.1±0.2	4.7±0.2	4.6±0.3	1.8±0.1	1.8±0.1	11.9±0.2	-6.9±0.2
3	-7.0±0.2	-2.6±0.2	2.3±0.3	-4.3±0.2	1.8±0.2	5.7±0.3	3.1±0.1	2.2±0.1	11.2±0.2	-6.8±0.2
4	-3.6±0.2	-2.3±0.2	-1.8±0.3	0.5±0.2	0.9±0.2	4.0±0.3	2.2±0.1	2.4±0.1	9.7±0.2	-7.4±0.2
5	-3.2±0.2	0.0±0.2	-4.4±0.4	-2.8±0.2	0.1±0.2	4.7±0.3	1.7±0.1	3.3±0.1	10.7±0.2	-6.2±0.2
**Av**	**-2.7±1.3**	**-1.6±0.6**	**-2.1±1.2**	**-1.4±0.9**	**1.1±1.1**	**5.0±0.4**	**2.0±0.3**	**2.3±0.3**	**11.1±0.4**	**-6.5±0.4**
Err	-5.8	1.0	-1.6	-3.6	-2.0	7.4	-0.3	-0.8	2.3	-2.4
Qual	2.7±0.2	7.4±0.5	0.46±0.06	0.43±0.05						
SIS										
1	1.0±0.2	-3.3±0.2	1.0±0.3	-1.1±0.2	-2.1±0.2	3.7±0.3	0.2±0.1	2.0±0.1	10.2±0.2	-4.6±0.2
2	-4.2±0.2	-3.8±0.2	-2.4±0.3	0.2±0.2	-0.1±0.2	8.5±0.3	1.2±0.1	3.1±0.1	7.2±0.2	-5.4±0.2
3	-3.8±0.2	-0.2±0.2	2.2±0.4	2.7±0.2	0.5±0.2	4.6±0.3	1.8±0.1	3.0±0.1	10.0±0.2	-6.4±0.2
4	3.0±0.2	-4.3±0.2	-2.8±0.4	0.4±0.2	-1.5±0.2	3.7±0.3	1.0±0.1	2.4±0.1	13.8±0.2	-5.8±0.2
5	1.4±0.2	-2.8±0.2	-1.5±0.3	-0.6±0.2	-3.1±0.2	5.6±0.3	0.1±0.1	2.6±0.1	13.0±0.2	-5.4±0.2
**Av**	**-0.5±1.5**	**-2.9±0.7**	**-0.7±1.0**	**0.3±0.7**	**-1.3±0.7**	**5.2±0.9**	**0.9±0.3**	**2.6±0.2**	**10.9±1.2**	**-5.5±0.3**
Err	-3.6	-0.3	-0.3	-1.9	-4.4	7.6	-1.4	-0.5	2.1	-1.4
Qual	2.3±0.3	7.6±0.9	0.50±0.07	0.43±0.05						

Table S8. AMBER topology (.off) files for the four BRD ligands and the seven T4 lysozyme ligands. The second and last column of the first entry of each ligand contain the AMBER atom types and charges of the ligands.

```
!!index array str
 "L1"
!entry.L1.unit.atoms table  str name  str type  int typex  int resx  int flags  int seq  int
elmnt  dbl chg
 "C1" "ca" 0 1 131072 1 6 -0.186000
 "C2" "ca" 0 1 131072 2 6 -0.096500
 "C3" "ca" 0 1 131072 3 6 -0.168000
 "C4" "ca" 0 1 131072 4 6 -0.096500
 "C5" "ca" 0 1 131072 5 6 -0.186000
 "C6" "ca" 0 1 131072 6 6 0.140600
 "N1" "nu" 0 1 131072 7 7 -0.715600
 "C7" "c6" 0 1 131072 8 6 0.247800
 "C8" "ca" 0 1 131072 9 6 -0.113300
 "C9" "ca" 0 1 131072 10 6 -0.097000
 "C10" "c6" 0 1 131072 11 6 -0.124400
 "C11" "c6" 0 1 131072 12 6 0.117700
 "C12" "c3" 0 1 131072 13 6 -0.114100
 "N2" "n" 0 1 131072 14 7 -0.391000
 "C13" "c" 0 1 131072 15 6 0.668100
 "C14" "c3" 0 1 131072 16 6 -0.171100
 "O1" "o" 0 1 131072 17 8 -0.589100
 "C15" "ca" 0 1 131072 18 6 0.063600
 "C16" "ca" 0 1 131072 19 6 -0.126000
 "C17" "ca" 0 1 131072 20 6 -0.106000
 "C18" "cp" 0 1 131072 21 6 -0.046000
 "C19" "cp" 0 1 131072 22 6 -0.070000
 "C20" "ca" 0 1 131072 23 6 -0.086500
 "C21" "ca" 0 1 131072 24 6 -0.180500
 "C22" "ca" 0 1 131072 25 6 0.125100
 "O2" "os" 0 1 131072 26 8 -0.326900
 "C23" "c3" 0 1 131072 27 6 0.113700
 "C24" "ca" 0 1 131072 28 6 -0.180500
 "C25" "ca" 0 1 131072 29 6 -0.086500
 "H1" "ha" 0 1 131072 30 1 0.131500
 "H2" "ha" 0 1 131072 31 1 0.130000
 "H3" "ha" 0 1 131072 32 1 0.132000
 "H4" "ha" 0 1 131072 33 1 0.130000
 "H5" "ha" 0 1 131072 34 1 0.131500
 "H6" "hn" 0 1 131072 35 1 0.392800
 "H7" "ha" 0 1 131072 36 1 0.146000
 "H8" "h1" 0 1 131072 37 1 0.078700
 "H9" "hc" 0 1 131072 38 1 0.060200
 "H10" "hc" 0 1 131072 39 1 0.060200
 "H11" "hc" 0 1 131072 40 1 0.046033
 "H12" "hc" 0 1 131072 41 1 0.046033
 "H13" "hc" 0 1 131072 42 1 0.046033
 "H14" "h1" 0 1 131072 43 1 0.048700
 "H15" "hc" 0 1 131072 44 1 0.067700
 "H16" "hc" 0 1 131072 45 1 0.067700
 "H17" "hc" 0 1 131072 46 1 0.067700
 "H18" "ha" 0 1 131072 47 1 0.162000
 "H19" "ha" 0 1 131072 48 1 0.138000
 "H20" "ha" 0 1 131072 49 1 0.138000
 "H21" "ha" 0 1 131072 50 1 0.143500
 "H22" "h1" 0 1 131072 51 1 0.044367
 "H23" "h1" 0 1 131072 52 1 0.044367
 "H24" "h1" 0 1 131072 53 1 0.044367
 "H25" "ha" 0 1 131072 54 1 0.143500
 "H26" "ha" 0 1 131072 55 1 0.138000
!entry.L1.unit.atomspertinfo table  str pname  str ptype  int ptypex  int pelmnt  dbl pchg
 "C1" "ca" 0 -1 0.0
 "C2" "ca" 0 -1 0.0
 "C3" "ca" 0 -1 0.0
 "C4" "ca" 0 -1 0.0
 "C5" "ca" 0 -1 0.0
```

```
 "C6" "ca" 0 -1 0.0
 "N1" "nu" 0 -1 0.0
 "C7" "c6" 0 -1 0.0
 "C8" "ca" 0 -1 0.0
 "C9" "ca" 0 -1 0.0
 "C10" "c6" 0 -1 0.0
 "C11" "c6" 0 -1 0.0
 "C12" "c3" 0 -1 0.0
 "N2" "n" 0 -1 0.0
 "C13" "c" 0 -1 0.0
 "C14" "c3" 0 -1 0.0
 "O1" "o" 0 -1 0.0
 "C15" "ca" 0 -1 0.0
 "C16" "ca" 0 -1 0.0
 "C17" "ca" 0 -1 0.0
 "C18" "cp" 0 -1 0.0
 "C19" "cp" 0 -1 0.0
 "C20" "ca" 0 -1 0.0
 "C21" "ca" 0 -1 0.0
 "C22" "ca" 0 -1 0.0
 "O2" "os" 0 -1 0.0
 "C23" "c3" 0 -1 0.0
 "C24" "ca" 0 -1 0.0
 "C25" "ca" 0 -1 0.0
 "H1" "ha" 0 -1 0.0
 "H2" "ha" 0 -1 0.0
 "H3" "ha" 0 -1 0.0
 "H4" "ha" 0 -1 0.0
 "H5" "ha" 0 -1 0.0
 "H6" "hn" 0 -1 0.0
 "H7" "ha" 0 -1 0.0
 "H8" "h1" 0 -1 0.0
 "H9" "hc" 0 -1 0.0
 "H10" "hc" 0 -1 0.0
 "H11" "hc" 0 -1 0.0
 "H12" "hc" 0 -1 0.0
 "H13" "hc" 0 -1 0.0
 "H14" "h1" 0 -1 0.0
 "H15" "hc" 0 -1 0.0
 "H16" "hc" 0 -1 0.0
 "H17" "hc" 0 -1 0.0
 "H18" "ha" 0 -1 0.0
 "H19" "ha" 0 -1 0.0
 "H20" "ha" 0 -1 0.0
 "H21" "ha" 0 -1 0.0
 "H22" "h1" 0 -1 0.0
 "H23" "h1" 0 -1 0.0
 "H24" "h1" 0 -1 0.0
 "H25" "ha" 0 -1 0.0
 "H26" "ha" 0 -1 0.0
!entry.L1.unit.boundbox array dbl
 -1.000000
 0.0
 0.0
 0.0
 0.0
!entry.L1.unit.childsequence single int
 2
!entry.L1.unit.connect array int
 0
 0
!entry.L1.unit.connectivity table  int atom1x  int atom2x  int flags
 1 2 4
 1 6 4
 1 30 1
 2 3 4
 2 31 1
 3 4 4
 3 32 1
 4 5 4
 4 33 1
```

```
 5 6 4
 5 34 1
 6 7 1
 7 8 1
 7 35 1
 8 9 1
 8 11 1
 8 37 1
 9 10 4
 9 18 4
 10 21 4
 10 36 1
 11 12 1
 11 38 1
 11 39 1
 12 13 1
 12 14 1
 12 43 1
 13 40 1
 13 41 1
 13 42 1
 14 15 1
 14 18 1
 15 16 1
 15 17 2
 16 44 1
 16 45 1
 16 46 1
 18 19 4
 19 20 4
 19 47 1
 20 21 4
 20 48 1
 21 22 1
 22 23 4
 22 29 4
 23 24 4
 23 49 1
 24 25 4
 24 50 1
 25 26 1
 25 28 4
 26 27 1
 27 51 1
 27 52 1
 27 53 1
 28 29 4
 28 54 1
 29 55 1
!entry.L1.unit.hierarchy table  str abovetype  int abovex  str belowtype  int belowx
 "U" 0 "R" 1
 "R" 1 "A" 1
 "R" 1 "A" 2
 "R" 1 "A" 3
 "R" 1 "A" 4
 "R" 1 "A" 5
 "R" 1 "A" 6
 "R" 1 "A" 7
 "R" 1 "A" 8
 "R" 1 "A" 9
 "R" 1 "A" 10
 "R" 1 "A" 11
 "R" 1 "A" 12
 "R" 1 "A" 13
 "R" 1 "A" 14
 "R" 1 "A" 15
 "R" 1 "A" 16
 "R" 1 "A" 17
 "R" 1 "A" 18
 "R" 1 "A" 19
 "R" 1 "A" 20
```

```
 "R" 1 "A" 21
 "R" 1 "A" 22
 "R" 1 "A" 23
 "R" 1 "A" 24
 "R" 1 "A" 25
 "R" 1 "A" 26
 "R" 1 "A" 27
 "R" 1 "A" 28
 "R" 1 "A" 29
 "R" 1 "A" 30
 "R" 1 "A" 31
 "R" 1 "A" 32
 "R" 1 "A" 33
 "R" 1 "A" 34
 "R" 1 "A" 35
 "R" 1 "A" 36
 "R" 1 "A" 37
 "R" 1 "A" 38
 "R" 1 "A" 39
 "R" 1 "A" 40
 "R" 1 "A" 41
 "R" 1 "A" 42
 "R" 1 "A" 43
 "R" 1 "A" 44
 "R" 1 "A" 45
 "R" 1 "A" 46
 "R" 1 "A" 47
 "R" 1 "A" 48
 "R" 1 "A" 49
 "R" 1 "A" 50
 "R" 1 "A" 51
 "R" 1 "A" 52
 "R" 1 "A" 53
 "R" 1 "A" 54
 "R" 1 "A" 55
!entry.L1.unit.name single str
 "L1"
!entry.L1.unit.positions table  dbl x  dbl y  dbl z
 -1.119000 2.963000 -0.993000
 -0.838000 4.115000 -1.729000
 -0.767000 5.365000 -1.113000
 -0.982000 5.450000 0.265000
 -1.270000 4.311000 1.011000
 -1.345000 3.047000 0.393000
 -1.604000 1.917000 1.170000
 -2.166000 0.695000 0.614000
 -1.134000 -0.347000 0.216000
 0.240000 -0.136000 0.266000
 -3.096000 0.017000 1.632000
 -3.777000 -1.251000 1.053000
 -3.912000 -2.338000 2.130000
 -3.056000 -1.763000 -0.141000
 -3.708000 -2.388000 -1.199000
 -5.231000 -2.457000 -1.151000
 -3.100000 -2.851000 -2.153000
 -1.635000 -1.604000 -0.160000
 -0.749000 -2.627000 -0.509000
 0.623000 -2.391000 -0.484000
 1.148000 -1.147000 -0.092000
 2.611000 -0.904000 -0.057000
 3.150000 0.353000 -0.394000
 4.517000 0.586000 -0.364000
 5.401000 -0.438000 0.006000
 6.727000 -0.110000 0.003000
 7.666000 -1.113000 0.355000
 4.890000 -1.695000 0.347000
 3.511000 -1.913000 0.313000
 -1.141000 2.000000 -1.492000
 -0.667000 4.026000 -2.799000
 -0.545000 6.256000 -1.693000
 -0.932000 6.413000 0.767000
```

-1.440000 4.391000 2.083000
-1.906000 2.126000 2.112000
0.602000 0.827000 0.611000
-2.766000 0.949000 -0.276000
-3.873000 0.714000 1.972000
-2.493000 -0.253000 2.509000
-4.439000 -1.945000 3.008000
-4.465000 -3.206000 1.760000
-2.920000 -2.677000 2.450000
-4.772000 -0.960000 0.713000
-5.546000 -3.053000 -2.008000
-5.611000 -2.919000 -0.235000
-5.681000 -1.460000 -1.235000
-1.133000 -3.588000 -0.827000
1.298000 -3.182000 -0.796000
2.485000 1.153000 -0.708000
4.928000 1.553000 -0.635000
7.616000 -1.971000 -0.328000
7.516000 -1.464000 1.385000
8.649000 -0.644000 0.275000
5.547000 -2.504000 0.648000
3.131000 -2.887000 0.606000
!entry.L1.unit.residueconnect table int c1x int c2x int c3x int c4x int c5x int c6x
0 0 0 0 0 0
!entry.L1.unit.residues table str name int seq int childseq int startatomx str restype int imagingx
"L1" 1 56 1 "?" 0
!entry.L1.unit.residuesPdbSequenceNumber array int
0
!entry.L1.unit.solventcap array dbl
-1.000000
0.0
0.0
0.0
0.0
!entry.L1.unit.velocities table dbl x dbl y dbl z
0.0 0.0 0.0
0.0 0.0 0.0
0.0 0.0 0.0
0.0 0.0 0.0
0.0 0.0 0.0
0.0 0.0 0.0
0.0 0.0 0.0
0.0 0.0 0.0
0.0 0.0 0.0
0.0 0.0 0.0
0.0 0.0 0.0
0.0 0.0 0.0
0.0 0.0 0.0
0.0 0.0 0.0
0.0 0.0 0.0
0.0 0.0 0.0
0.0 0.0 0.0
0.0 0.0 0.0
0.0 0.0 0.0
0.0 0.0 0.0
0.0 0.0 0.0
0.0 0.0 0.0
0.0 0.0 0.0
0.0 0.0 0.0
0.0 0.0 0.0
0.0 0.0 0.0
0.0 0.0 0.0
0.0 0.0 0.0
0.0 0.0 0.0
0.0 0.0 0.0
0.0 0.0 0.0
0.0 0.0 0.0
0.0 0.0 0.0
0.0 0.0 0.0
0.0 0.0 0.0

```
 0.0 0.0 0.0
 0.0 0.0 0.0
 0.0 0.0 0.0
 0.0 0.0 0.0
 0.0 0.0 0.0
 0.0 0.0 0.0
 0.0 0.0 0.0
 0.0 0.0 0.0
 0.0 0.0 0.0
 0.0 0.0 0.0
 0.0 0.0 0.0
 0.0 0.0 0.0
 0.0 0.0 0.0
 0.0 0.0 0.0
 0.0 0.0 0.0
 0.0 0.0 0.0
 0.0 0.0 0.0
 0.0 0.0 0.0
 0.0 0.0 0.0
 0.0 0.0 0.0


!!index array str
 "L2"
!entry.L2.unit.atoms table  str name  str type  int typex  int resx  int flags  int seq  int
elmnt  dbl chg
 "C1" "ca" 0 1 131072 1 6 -0.186000
 "C2" "ca" 0 1 131072 2 6 -0.096500
 "C3" "ca" 0 1 131072 3 6 -0.168000
 "C4" "ca" 0 1 131072 4 6 -0.096500
 "C5" "ca" 0 1 131072 5 6 -0.186000
 "C6" "ca" 0 1 131072 6 6 0.140600
 "N1" "nu" 0 1 131072 7 7 -0.714600
 "C7" "c6" 0 1 131072 8 6 0.247800
 "C8" "ca" 0 1 131072 9 6 -0.113300
 "C9" "ca" 0 1 131072 10 6 -0.105000
 "C10" "c6" 0 1 131072 11 6 -0.124400
 "C11" "c6" 0 1 131072 12 6 0.118700
 "C12" "c3" 0 1 131072 13 6 -0.114100
 "N2" "n" 0 1 131072 14 7 -0.391000
 "C13" "c" 0 1 131072 15 6 0.668100
 "C14" "c3" 0 1 131072 16 6 -0.171100
 "O1" "o" 0 1 131072 17 8 -0.590100
 "C15" "ca" 0 1 131072 18 6 0.062600
 "C16" "ca" 0 1 131072 19 6 -0.125000
 "C17" "ca" 0 1 131072 20 6 -0.115000
 "C18" "ca" 0 1 131072 21 6 -0.146000
 "H1" "ha" 0 1 131072 22 1 0.133000
 "H2" "ha" 0 1 131072 23 1 0.131000
 "H3" "ha" 0 1 131072 24 1 0.129500
 "H4" "ha" 0 1 131072 25 1 0.132000
 "H5" "ha" 0 1 131072 26 1 0.129500
 "H6" "ha" 0 1 131072 27 1 0.131000
 "H7" "hn" 0 1 131072 28 1 0.391800
 "H8" "ha" 0 1 131072 29 1 0.142000
 "H9" "h1" 0 1 131072 30 1 0.078700
 "H10" "hc" 0 1 131072 31 1 0.060200
 "H11" "hc" 0 1 131072 32 1 0.060200
 "H12" "hc" 0 1 131072 33 1 0.046033
 "H13" "hc" 0 1 131072 34 1 0.046033
 "H14" "hc" 0 1 131072 35 1 0.046033
 "H15" "h1" 0 1 131072 36 1 0.048700
 "H16" "hc" 0 1 131072 37 1 0.067700
 "H17" "hc" 0 1 131072 38 1 0.067700
 "H18" "hc" 0 1 131072 39 1 0.067700
 "H19" "ha" 0 1 131072 40 1 0.161000
 "H20" "ha" 0 1 131072 41 1 0.135000
!entry.L2.unit.atomspertinfo table  str pname  str ptype  int ptypex  int pelmnt  dbl pchg
 "C1" "ca" 0 -1 0.0
 "C2" "ca" 0 -1 0.0
```

```
 "C3" "ca" 0 -1 0.0
 "C4" "ca" 0 -1 0.0
 "C5" "ca" 0 -1 0.0
 "C6" "ca" 0 -1 0.0
 "N1" "nu" 0 -1 0.0
 "C7" "c6" 0 -1 0.0
 "C8" "ca" 0 -1 0.0
 "C9" "ca" 0 -1 0.0
 "C10" "c6" 0 -1 0.0
 "C11" "c6" 0 -1 0.0
 "C12" "c3" 0 -1 0.0
 "N2" "n" 0 -1 0.0
 "C13" "c" 0 -1 0.0
 "C14" "c3" 0 -1 0.0
 "O1" "o" 0 -1 0.0
 "C15" "ca" 0 -1 0.0
 "C16" "ca" 0 -1 0.0
 "C17" "ca" 0 -1 0.0
 "C18" "ca" 0 -1 0.0
 "H1" "ha" 0 -1 0.0
 "H2" "ha" 0 -1 0.0
 "H3" "ha" 0 -1 0.0
 "H4" "ha" 0 -1 0.0
 "H5" "ha" 0 -1 0.0
 "H6" "ha" 0 -1 0.0
 "H7" "hn" 0 -1 0.0
 "H8" "ha" 0 -1 0.0
 "H9" "h1" 0 -1 0.0
 "H10" "hc" 0 -1 0.0
 "H11" "hc" 0 -1 0.0
 "H12" "hc" 0 -1 0.0
 "H13" "hc" 0 -1 0.0
 "H14" "hc" 0 -1 0.0
 "H15" "h1" 0 -1 0.0
 "H16" "hc" 0 -1 0.0
 "H17" "hc" 0 -1 0.0
 "H18" "hc" 0 -1 0.0
 "H19" "ha" 0 -1 0.0
 "H20" "ha" 0 -1 0.0
!entry.L2.unit.boundbox array dbl
 -1.000000
 0.0
 0.0
 0.0
 0.0
!entry.L2.unit.childsequence single int
 2
!entry.L2.unit.connect array int
 0
 0
!entry.L2.unit.connectivity table  int atom1x  int atom2x  int flags
 1 2 4
 1 6 4
 1 23 1
 2 3 4
 2 24 1
 3 4 4
 3 25 1
 4 5 4
 4 26 1
 5 6 4
 5 27 1
 6 7 1
 7 8 1
 7 28 1
 8 9 1
 8 11 1
 8 30 1
 9 10 4
 9 18 4
 10 21 4
```

```
 10 29 1
 11 12 1
 11 31 1
 11 32 1
 12 13 1
 12 14 1
 12 36 1
 13 33 1
 13 34 1
 13 35 1
 14 15 1
 14 18 1
 15 16 1
 15 17 2
 16 37 1
 16 38 1
 16 39 1
 18 19 4
 19 20 4
 19 40 1
 20 21 4
 20 41 1
 21 22 1
!entry.L2.unit.hierarchy table  str abovetype  int abovex  str belowtype  int belowx
 "U" 0 "R" 1
 "R" 1 "A" 1
 "R" 1 "A" 2
 "R" 1 "A" 3
 "R" 1 "A" 4
 "R" 1 "A" 5
 "R" 1 "A" 6
 "R" 1 "A" 7
 "R" 1 "A" 8
 "R" 1 "A" 9
 "R" 1 "A" 10
 "R" 1 "A" 11
 "R" 1 "A" 12
 "R" 1 "A" 13
 "R" 1 "A" 14
 "R" 1 "A" 15
 "R" 1 "A" 16
 "R" 1 "A" 17
 "R" 1 "A" 18
 "R" 1 "A" 19
 "R" 1 "A" 20
 "R" 1 "A" 21
 "R" 1 "A" 22
 "R" 1 "A" 23
 "R" 1 "A" 24
 "R" 1 "A" 25
 "R" 1 "A" 26
 "R" 1 "A" 27
 "R" 1 "A" 28
 "R" 1 "A" 29
 "R" 1 "A" 30
 "R" 1 "A" 31
 "R" 1 "A" 32
 "R" 1 "A" 33
 "R" 1 "A" 34
 "R" 1 "A" 35
 "R" 1 "A" 36
 "R" 1 "A" 37
 "R" 1 "A" 38
 "R" 1 "A" 39
 "R" 1 "A" 40
 "R" 1 "A" 41
!entry.L2.unit.name single str
 "L2"
!entry.L2.unit.positions table  dbl x  dbl y  dbl z
 2.815000 0.152000 0.994000
 3.991000 0.242000 1.738000
```

```
 5.208000 -0.200000 1.219000
 5.235000 -0.737000 -0.070000
 4.069000 -0.839000 -0.822000
 2.837000 -0.397000 -0.301000
 1.687000 -0.482000 -1.088000
 0.349000 -0.529000 -0.515000
 -0.350000 0.819000 -0.445000
 0.254000 2.031000 -0.777000
 -0.561000 -1.446000 -1.348000
 -1.978000 -1.582000 -0.731000
 -3.046000 -1.664000 -1.831000
 -2.268000 -0.483000 0.223000
 -3.061000 -0.661000 1.352000
 -3.564000 -2.071000 1.648000
 -3.337000 0.262000 2.105000
 -1.703000 0.799000 -0.067000
 -2.437000 1.988000 -0.006000
 -1.813000 3.195000 -0.320000
 -0.472000 3.221000 -0.708000
 0.007000 4.164000 -0.956000
 1.886000 0.530000 1.410000
 3.950000 0.670000 2.737000
 6.120000 -0.124000 1.805000
 6.172000 -1.088000 -0.496000
 4.102000 -1.264000 -1.823000
 1.803000 -1.042000 -1.922000
 1.295000 2.031000 -1.086000
 0.410000 -0.947000 0.504000
 -0.117000 -2.444000 -1.445000
 -0.634000 -1.020000 -2.357000
 -2.815000 -2.475000 -2.532000
 -4.041000 -1.849000 -1.413000
 -3.083000 -0.725000 -2.393000
 -1.992000 -2.507000 -0.151000
 -4.225000 -1.994000 2.512000
 -4.117000 -2.514000 0.813000
 -2.739000 -2.748000 1.899000
 -3.474000 1.965000 0.301000
 -2.382000 4.119000 -0.264000
!entry.L2.unit.residueconnect table  int c1x  int c2x  int c3x  int c4x  int c5x  int c6x
 0 0 0 0 0 0
!entry.L2.unit.residues table  str name  int seq  int childseq  int startatomx  str restype  int
imagingx
 "L2" 1 42 1 "?" 0
!entry.L2.unit.residuesPdbSequenceNumber array int
 0
!entry.L2.unit.solventcap array dbl
 -1.000000
 0.0
 0.0
 0.0
 0.0
!entry.L2.unit.velocities table  dbl x  dbl y  dbl z
 0.0 0.0 0.0
 0.0 0.0 0.0
 0.0 0.0 0.0
 0.0 0.0 0.0
 0.0 0.0 0.0
 0.0 0.0 0.0
 0.0 0.0 0.0
 0.0 0.0 0.0
 0.0 0.0 0.0
 0.0 0.0 0.0
 0.0 0.0 0.0
 0.0 0.0 0.0
 0.0 0.0 0.0
 0.0 0.0 0.0
 0.0 0.0 0.0
 0.0 0.0 0.0
 0.0 0.0 0.0
 0.0 0.0 0.0
```

```
 0.0 0.0 0.0
 0.0 0.0 0.0
 0.0 0.0 0.0
 0.0 0.0 0.0
 0.0 0.0 0.0
 0.0 0.0 0.0
 0.0 0.0 0.0
 0.0 0.0 0.0
 0.0 0.0 0.0
 0.0 0.0 0.0
 0.0 0.0 0.0
 0.0 0.0 0.0
 0.0 0.0 0.0
 0.0 0.0 0.0
 0.0 0.0 0.0
 0.0 0.0 0.0
 0.0 0.0 0.0
 0.0 0.0 0.0
 0.0 0.0 0.0
 0.0 0.0 0.0
 0.0 0.0 0.0
 0.0 0.0 0.0
 0.0 0.0 0.0
```

```
!!index array str
 "L3"
!entry.L3.unit.atoms table  str name  str type  int typex  int resx  int flags  int seq  int
elmnt  dbl chg
 "C1" "ca" 0 1 131072 1 6 -0.185500
 "C2" "ca" 0 1 131072 2 6 -0.096500
 "C3" "ca" 0 1 131072 3 6 -0.168000
 "C4" "ca" 0 1 131072 4 6 -0.096500
 "C5" "ca" 0 1 131072 5 6 -0.185500
 "C6" "ca" 0 1 131072 6 6 0.140600
 "N1" "nu" 0 1 131072 7 7 -0.715600
 "C7" "c6" 0 1 131072 8 6 0.247800
 "C8" "ca" 0 1 131072 9 6 -0.114300
 "C9" "ca" 0 1 131072 10 6 -0.094000
 "C10" "c6" 0 1 131072 11 6 -0.124400
 "C11" "c6" 0 1 131072 12 6 0.117700
 "C12" "c3" 0 1 131072 13 6 -0.114100
 "N2" "n" 0 1 131072 14 7 -0.391000
 "C13" "c" 0 1 131072 15 6 0.668100
 "C14" "c3" 0 1 131072 16 6 -0.171100
 "O1" "o" 0 1 131072 17 8 -0.589100
 "C15" "ca" 0 1 131072 18 6 0.065600
 "C16" "ca" 0 1 131072 19 6 -0.127000
 "C17" "ca" 0 1 131072 20 6 -0.103000
 "C18" "cp" 0 1 131072 21 6 -0.054000
 "C19" "cp" 0 1 131072 22 6 -0.035000
 "C20" "ca" 0 1 131072 23 6 -0.120000
 "C21" "ca" 0 1 131072 24 6 -0.130500
 "C22" "ca" 0 1 131072 25 6 -0.129000
 "C23" "ca" 0 1 131072 26 6 -0.130500
 "C24" "ca" 0 1 131072 27 6 -0.120000
 "H1" "ha" 0 1 131072 28 1 0.131000
 "H2" "ha" 0 1 131072 29 1 0.131500
 "H3" "ha" 0 1 131072 30 1 0.130000
 "H4" "ha" 0 1 131072 31 1 0.132000
 "H5" "ha" 0 1 131072 32 1 0.130000
 "H6" "ha" 0 1 131072 33 1 0.131500
 "H7" "hn" 0 1 131072 34 1 0.392800
 "H8" "ha" 0 1 131072 35 1 0.147000
 "H9" "h1" 0 1 131072 36 1 0.078700
 "H10" "hc" 0 1 131072 37 1 0.060200
 "H11" "hc" 0 1 131072 38 1 0.060200
 "H12" "hc" 0 1 131072 39 1 0.046033
 "H13" "hc" 0 1 131072 40 1 0.046033
 "H14" "hc" 0 1 131072 41 1 0.046033
```

```
 "H15" "h1" 0 1 131072 42 1 0.048700
 "H16" "hc" 0 1 131072 43 1 0.068367
 "H17" "hc" 0 1 131072 44 1 0.068367
 "H18" "hc" 0 1 131072 45 1 0.068367
 "H19" "ha" 0 1 131072 46 1 0.162000
 "H20" "ha" 0 1 131072 47 1 0.140000
 "H21" "ha" 0 1 131072 48 1 0.136000
 "H22" "ha" 0 1 131072 49 1 0.131500
 "H23" "ha" 0 1 131072 50 1 0.131500
 "H24" "ha" 0 1 131072 51 1 0.136000
!entry.L3.unit.atomspertinfo table  str pname  str ptype  int ptypex  int pelmnt  dbl pchg
 "C1" "ca" 0 -1 0.0
 "C2" "ca" 0 -1 0.0
 "C3" "ca" 0 -1 0.0
 "C4" "ca" 0 -1 0.0
 "C5" "ca" 0 -1 0.0
 "C6" "ca" 0 -1 0.0
 "N1" "nu" 0 -1 0.0
 "C7" "c6" 0 -1 0.0
 "C8" "ca" 0 -1 0.0
 "C9" "ca" 0 -1 0.0
 "C10" "c6" 0 -1 0.0
 "C11" "c6" 0 -1 0.0
 "C12" "c3" 0 -1 0.0
 "N2" "n" 0 -1 0.0
 "C13" "c" 0 -1 0.0
 "C14" "c3" 0 -1 0.0
 "O1" "o" 0 -1 0.0
 "C15" "ca" 0 -1 0.0
 "C16" "ca" 0 -1 0.0
 "C17" "ca" 0 -1 0.0
 "C18" "cp" 0 -1 0.0
 "C19" "cp" 0 -1 0.0
 "C20" "ca" 0 -1 0.0
 "C21" "ca" 0 -1 0.0
 "C22" "ca" 0 -1 0.0
 "C23" "ca" 0 -1 0.0
 "C24" "ca" 0 -1 0.0
 "H1" "ha" 0 -1 0.0
 "H2" "ha" 0 -1 0.0
 "H3" "ha" 0 -1 0.0
 "H4" "ha" 0 -1 0.0
 "H5" "ha" 0 -1 0.0
 "H6" "ha" 0 -1 0.0
 "H7" "hn" 0 -1 0.0
 "H8" "ha" 0 -1 0.0
 "H9" "h1" 0 -1 0.0
 "H10" "hc" 0 -1 0.0
 "H11" "hc" 0 -1 0.0
 "H12" "hc" 0 -1 0.0
 "H13" "hc" 0 -1 0.0
 "H14" "hc" 0 -1 0.0
 "H15" "h1" 0 -1 0.0
 "H16" "hc" 0 -1 0.0
 "H17" "hc" 0 -1 0.0
 "H18" "hc" 0 -1 0.0
 "H19" "ha" 0 -1 0.0
 "H20" "ha" 0 -1 0.0
 "H21" "ha" 0 -1 0.0
 "H22" "ha" 0 -1 0.0
 "H23" "ha" 0 -1 0.0
 "H24" "ha" 0 -1 0.0
!entry.L3.unit.boundbox array dbl
 -1.000000
 0.0
 0.0
 0.0
 0.0
!entry.L3.unit.childsequence single int
 2
!entry.L3.unit.connect array int
```

```
 0
 0
!entry.L3.unit.connectivity table  int atom1x  int atom2x  int flags
 1 2 4
 1 6 4
 1 29 1
 2 3 4
 2 30 1
 3 4 4
 3 31 1
 4 5 4
 4 32 1
 5 6 4
 5 33 1
 6 7 1
 7 8 1
 7 34 1
 8 9 1
 8 11 1
 8 36 1
 9 10 4
 9 18 4
 10 21 4
 10 35 1
 11 12 1
 11 37 1
 11 38 1
 12 13 1
 12 14 1
 12 42 1
 13 39 1
 13 40 1
 13 41 1
 14 15 1
 14 18 1
 15 16 1
 15 17 2
 16 43 1
 16 44 1
 16 45 1
 18 19 4
 19 20 4
 19 46 1
 20 21 4
 20 47 1
 21 22 1
 22 23 4
 22 27 4
 23 24 4
 23 48 1
 24 25 4
 24 49 1
 25 26 4
 25 28 1
 26 27 4
 26 50 1
 27 51 1
!entry.L3.unit.hierarchy table  str abovetype  int abovex  str belowtype  int belowx
 "U" 0 "R" 1
 "R" 1 "A" 1
 "R" 1 "A" 2
 "R" 1 "A" 3
 "R" 1 "A" 4
 "R" 1 "A" 5
 "R" 1 "A" 6
 "R" 1 "A" 7
 "R" 1 "A" 8
 "R" 1 "A" 9
 "R" 1 "A" 10
 "R" 1 "A" 11
 "R" 1 "A" 12
```

```
 "R" 1 "A" 13
 "R" 1 "A" 14
 "R" 1 "A" 15
 "R" 1 "A" 16
 "R" 1 "A" 17
 "R" 1 "A" 18
 "R" 1 "A" 19
 "R" 1 "A" 20
 "R" 1 "A" 21
 "R" 1 "A" 22
 "R" 1 "A" 23
 "R" 1 "A" 24
 "R" 1 "A" 25
 "R" 1 "A" 26
 "R" 1 "A" 27
 "R" 1 "A" 28
 "R" 1 "A" 29
 "R" 1 "A" 30
 "R" 1 "A" 31
 "R" 1 "A" 32
 "R" 1 "A" 33
 "R" 1 "A" 34
 "R" 1 "A" 35
 "R" 1 "A" 36
 "R" 1 "A" 37
 "R" 1 "A" 38
 "R" 1 "A" 39
 "R" 1 "A" 40
 "R" 1 "A" 41
 "R" 1 "A" 42
 "R" 1 "A" 43
 "R" 1 "A" 44
 "R" 1 "A" 45
 "R" 1 "A" 46
 "R" 1 "A" 47
 "R" 1 "A" 48
 "R" 1 "A" 49
 "R" 1 "A" 50
 "R" 1 "A" 51
!entry.L3.unit.name single str
 "L3"
!entry.L3.unit.positions table  dbl x  dbl y  dbl z
 0.306000 2.905000 -1.002000
 0.914000 3.922000 -1.739000
 1.316000 5.112000 -1.132000
 1.101000 5.274000 0.239000
 0.491000 4.272000 0.986000
 0.081000 3.070000 0.378000
 -0.500000 2.068000 1.157000
 -1.351000 1.031000 0.594000
 -0.619000 -0.251000 0.231000
 0.761000 -0.405000 0.310000
 -2.452000 0.636000 1.592000
 -3.423000 -0.422000 1.006000
 -3.859000 -1.420000 2.088000
 -2.835000 -1.120000 -0.165000
 -3.604000 -1.570000 -1.233000
 -5.092000 -1.234000 -1.226000
 -3.119000 -2.195000 -2.166000
 -1.422000 -1.338000 -0.150000
 -0.827000 -2.561000 -0.473000
 0.558000 -2.690000 -0.418000
 1.380000 -1.621000 -0.023000
 2.856000 -1.765000 0.040000
 3.701000 -0.697000 -0.309000
 5.087000 -0.830000 -0.251000
 5.663000 -2.036000 0.155000
 4.837000 -3.106000 0.504000
 3.451000 -2.972000 0.448000
 6.743000 -2.140000 0.199000
 0.028000 1.979000 -1.493000
```

```
 1.077000 3.773000 -2.804000
 1.790000 5.897000 -1.714000
 1.405000 6.193000 0.734000
 0.325000 4.413000 2.053000
 -0.761000 2.369000 2.086000
 1.356000 0.435000 0.655000
 -1.841000 1.421000 -0.313000
 -3.027000 1.518000 1.900000
 -1.963000 0.235000 2.490000
 -4.282000 -0.891000 2.951000
 -4.614000 -2.118000 1.714000
 -2.998000 -2.003000 2.433000
 -4.302000 0.111000 0.638000
 -5.532000 -1.734000 -2.089000
 -5.605000 -1.570000 -0.319000
 -5.260000 -0.154000 -1.325000
 -1.442000 -3.392000 -0.794000
 1.010000 -3.634000 -0.708000
 3.266000 0.238000 -0.651000
 5.719000 0.008000 -0.533000
 5.273000 -4.047000 0.829000
 2.819000 -3.803000 0.748000
!entry.L3.unit.residueconnect table  int c1x  int c2x  int c3x  int c4x  int c5x  int c6x
 0 0 0 0 0 0
!entry.L3.unit.residues table  str name  int seq  int childseq  int startatomx  str restype  int
imagingx
 "L3" 1 52 1 "?" 0
!entry.L3.unit.residuesPdbSequenceNumber array int
 0
!entry.L3.unit.solventcap array dbl
 -1.000000
 0.0
 0.0
 0.0
 0.0
!entry.L3.unit.velocities table  dbl x  dbl y  dbl z
 0.0 0.0 0.0
 0.0 0.0 0.0
 0.0 0.0 0.0
 0.0 0.0 0.0
 0.0 0.0 0.0
 0.0 0.0 0.0
 0.0 0.0 0.0
 0.0 0.0 0.0
 0.0 0.0 0.0
 0.0 0.0 0.0
 0.0 0.0 0.0
 0.0 0.0 0.0
 0.0 0.0 0.0
 0.0 0.0 0.0
 0.0 0.0 0.0
 0.0 0.0 0.0
 0.0 0.0 0.0
 0.0 0.0 0.0
 0.0 0.0 0.0
 0.0 0.0 0.0
 0.0 0.0 0.0
 0.0 0.0 0.0
 0.0 0.0 0.0
 0.0 0.0 0.0
 0.0 0.0 0.0
 0.0 0.0 0.0
 0.0 0.0 0.0
 0.0 0.0 0.0
 0.0 0.0 0.0
 0.0 0.0 0.0
 0.0 0.0 0.0
 0.0 0.0 0.0
 0.0 0.0 0.0
 0.0 0.0 0.0
 0.0 0.0 0.0
```

```
 0.0 0.0 0.0
 0.0 0.0 0.0
 0.0 0.0 0.0
 0.0 0.0 0.0
 0.0 0.0 0.0
 0.0 0.0 0.0
 0.0 0.0 0.0
 0.0 0.0 0.0
 0.0 0.0 0.0
 0.0 0.0 0.0
 0.0 0.0 0.0
 0.0 0.0 0.0
 0.0 0.0 0.0
 0.0 0.0 0.0
 0.0 0.0 0.0
 0.0 0.0 0.0


!!index array str
 "L4"
!entry.L4.unit.atoms table  str name  str type  int typex  int resx  int flags  int seq  int
elmnt  dbl chg
 "C1" "ca" 0 1 131072 1 6 -0.185000
 "C2" "ca" 0 1 131072 2 6 -0.096500
 "C3" "ca" 0 1 131072 3 6 -0.167000
 "C4" "ca" 0 1 131072 4 6 -0.096500
 "C5" "ca" 0 1 131072 5 6 -0.185000
 "C6" "ca" 0 1 131072 6 6 0.138600
 "N1" "nu" 0 1 131072 7 7 -0.714600
 "C7" "c6" 0 1 131072 8 6 0.246800
 "C8" "ca" 0 1 131072 9 6 -0.114300
 "C9" "ca" 0 1 131072 10 6 -0.093000
 "C10" "c6" 0 1 131072 11 6 -0.124400
 "C11" "c6" 0 1 131072 12 6 0.117700
 "C12" "c3" 0 1 131072 13 6 -0.114100
 "N2" "n" 0 1 131072 14 7 -0.392000
 "C13" "c" 0 1 131072 15 6 0.669100
 "C14" "c3" 0 1 131072 16 6 -0.171100
 "O1" "o" 0 1 131072 17 8 -0.588100
 "C15" "ca" 0 1 131072 18 6 0.069600
 "C16" "ca" 0 1 131072 19 6 -0.127000
 "C17" "ca" 0 1 131072 20 6 -0.101000
 "C18" "cp" 0 1 131072 21 6 -0.054000
 "C19" "cp" 0 1 131072 22 6 -0.146300
 "C20" "ca" 0 1 131072 23 6 -0.086000
 "C21" "ca" 0 1 131072 24 6 -0.245300
 "C22" "ca" 0 1 131072 25 6 0.391200
 "N3" "nb" 0 1 131072 26 7 -0.664000
 "C23" "ca" 0 1 131072 27 6 0.401200
 "H1" "h4" 0 1 131072 28 1 0.022100
 "H2" "ha" 0 1 131072 29 1 0.131500
 "H3" "ha" 0 1 131072 30 1 0.130500
 "H4" "ha" 0 1 131072 31 1 0.133000
 "H5" "ha" 0 1 131072 32 1 0.130500
 "H6" "ha" 0 1 131072 33 1 0.131500
 "H7" "hn" 0 1 131072 34 1 0.391800
 "H8" "ha" 0 1 131072 35 1 0.146000
 "H9" "h1" 0 1 131072 36 1 0.078700
 "H10" "hc" 0 1 131072 37 1 0.060700
 "H11" "hc" 0 1 131072 38 1 0.060700
 "H12" "hc" 0 1 131072 39 1 0.046367
 "H13" "hc" 0 1 131072 40 1 0.046367
 "H14" "hc" 0 1 131072 41 1 0.046367
 "H15" "h1" 0 1 131072 42 1 0.049700
 "H16" "hc" 0 1 131072 43 1 0.068700
 "H17" "hc" 0 1 131072 44 1 0.068700
 "H18" "hc" 0 1 131072 45 1 0.068700
 "H19" "ha" 0 1 131072 46 1 0.164000
 "H20" "ha" 0 1 131072 47 1 0.141000
 "H21" "ha" 0 1 131072 48 1 0.144000
```

```
 "H22" "ha" 0 1 131072 49 1 0.144000
 "H23" "h4" 0 1 131072 50 1 0.024100
!entry.L4.unit.atomspertinfo table  str pname  str ptype  int ptypex  int pelmnt  dbl pchg
 "C1" "ca" 0 -1 0.0
 "C2" "ca" 0 -1 0.0
 "C3" "ca" 0 -1 0.0
 "C4" "ca" 0 -1 0.0
 "C5" "ca" 0 -1 0.0
 "C6" "ca" 0 -1 0.0
 "N1" "nu" 0 -1 0.0
 "C7" "c6" 0 -1 0.0
 "C8" "ca" 0 -1 0.0
 "C9" "ca" 0 -1 0.0
 "C10" "c6" 0 -1 0.0
 "C11" "c6" 0 -1 0.0
 "C12" "c3" 0 -1 0.0
 "N2" "n" 0 -1 0.0
 "C13" "c" 0 -1 0.0
 "C14" "c3" 0 -1 0.0
 "O1" "o" 0 -1 0.0
 "C15" "ca" 0 -1 0.0
 "C16" "ca" 0 -1 0.0
 "C17" "ca" 0 -1 0.0
 "C18" "cp" 0 -1 0.0
 "C19" "cp" 0 -1 0.0
 "C20" "ca" 0 -1 0.0
 "C21" "ca" 0 -1 0.0
 "C22" "ca" 0 -1 0.0
 "N3" "nb" 0 -1 0.0
 "C23" "ca" 0 -1 0.0
 "H1" "h4" 0 -1 0.0
 "H2" "ha" 0 -1 0.0
 "H3" "ha" 0 -1 0.0
 "H4" "ha" 0 -1 0.0
 "H5" "ha" 0 -1 0.0
 "H6" "ha" 0 -1 0.0
 "H7" "hn" 0 -1 0.0
 "H8" "ha" 0 -1 0.0
 "H9" "h1" 0 -1 0.0
 "H10" "hc" 0 -1 0.0
 "H11" "hc" 0 -1 0.0
 "H12" "hc" 0 -1 0.0
 "H13" "hc" 0 -1 0.0
 "H14" "hc" 0 -1 0.0
 "H15" "h1" 0 -1 0.0
 "H16" "hc" 0 -1 0.0
 "H17" "hc" 0 -1 0.0
 "H18" "hc" 0 -1 0.0
 "H19" "ha" 0 -1 0.0
 "H20" "ha" 0 -1 0.0
 "H21" "ha" 0 -1 0.0
 "H22" "ha" 0 -1 0.0
 "H23" "h4" 0 -1 0.0
!entry.L4.unit.boundbox array dbl
 -1.000000
 0.0
 0.0
 0.0
 0.0
!entry.L4.unit.childsequence single int
 2
!entry.L4.unit.connect array int
 0
 0
!entry.L4.unit.connectivity table  int atom1x  int atom2x  int flags
 1 2 4
 1 6 4
 1 29 1
 2 3 4
 2 30 1
 3 4 4
```

```
 3 31 1
 4 5 4
 4 32 1
 5 6 4
 5 33 1
 6 7 1
 7 8 1
 7 34 1
 8 9 1
 8 11 1
 8 36 1
 9 10 4
 9 18 4
 10 21 4
 10 35 1
 11 12 1
 11 37 1
 11 38 1
 12 13 1
 12 14 1
 12 42 1
 13 39 1
 13 40 1
 13 41 1
 14 15 1
 14 18 1
 15 16 1
 15 17 2
 16 43 1
 16 44 1
 16 45 1
 18 19 4
 19 20 4
 19 46 1
 20 21 4
 20 47 1
 21 22 1
 22 23 4
 22 27 4
 23 24 4
 23 48 1
 24 25 4
 24 49 1
 25 26 4
 25 28 1
 26 27 4
 27 50 1
!entry.L4.unit.hierarchy table  str abovetype  int abovex  str belowtype  int belowx
 "U" 0 "R" 1
 "R" 1 "A" 1
 "R" 1 "A" 2
 "R" 1 "A" 3
 "R" 1 "A" 4
 "R" 1 "A" 5
 "R" 1 "A" 6
 "R" 1 "A" 7
 "R" 1 "A" 8
 "R" 1 "A" 9
 "R" 1 "A" 10
 "R" 1 "A" 11
 "R" 1 "A" 12
 "R" 1 "A" 13
 "R" 1 "A" 14
 "R" 1 "A" 15
 "R" 1 "A" 16
 "R" 1 "A" 17
 "R" 1 "A" 18
 "R" 1 "A" 19
 "R" 1 "A" 20
 "R" 1 "A" 21
 "R" 1 "A" 22
```

```
 "R" 1 "A" 23
 "R" 1 "A" 24
 "R" 1 "A" 25
 "R" 1 "A" 26
 "R" 1 "A" 27
 "R" 1 "A" 28
 "R" 1 "A" 29
 "R" 1 "A" 30
 "R" 1 "A" 31
 "R" 1 "A" 32
 "R" 1 "A" 33
 "R" 1 "A" 34
 "R" 1 "A" 35
 "R" 1 "A" 36
 "R" 1 "A" 37
 "R" 1 "A" 38
 "R" 1 "A" 39
 "R" 1 "A" 40
 "R" 1 "A" 41
 "R" 1 "A" 42
 "R" 1 "A" 43
 "R" 1 "A" 44
 "R" 1 "A" 45
 "R" 1 "A" 46
 "R" 1 "A" 47
 "R" 1 "A" 48
 "R" 1 "A" 49
 "R" 1 "A" 50
!entry.L4.unit.name single str
 "L4"
!entry.L4.unit.positions table  dbl x  dbl y  dbl z
 0.335000 2.900000 -1.005000
 0.956000 3.908000 -1.744000
 1.373000 5.093000 -1.139000
 1.160000 5.261000 0.232000
 0.537000 4.268000 0.980000
 0.112000 3.070000 0.374000
 -0.481000 2.077000 1.156000
 -1.341000 1.046000 0.594000
 -0.618000 -0.240000 0.230000
 0.762000 -0.401000 0.307000
 -2.443000 0.658000 1.593000
 -3.422000 -0.393000 1.007000
 -3.866000 -1.387000 2.090000
 -2.838000 -1.097000 -0.164000
 -3.611000 -1.545000 -1.231000
 -5.097000 -1.200000 -1.224000
 -3.128000 -2.173000 -2.162000
 -1.427000 -1.323000 -0.150000
 -0.840000 -2.549000 -0.476000
 0.545000 -2.687000 -0.423000
 1.371000 -1.622000 -0.027000
 2.844000 -1.772000 0.035000
 3.713000 -0.726000 -0.310000
 5.088000 -0.924000 -0.231000
 5.563000 -2.168000 0.187000
 4.759000 -3.182000 0.529000
 3.441000 -2.974000 0.454000
 6.632000 -2.360000 0.252000
 0.043000 1.977000 -1.495000
 1.117000 3.755000 -2.809000
 1.857000 5.871000 -1.722000
 1.476000 6.177000 0.725000
 0.373000 4.413000 2.047000
 -0.745000 2.385000 2.082000
 1.361000 0.434000 0.652000
 -1.830000 1.439000 -0.313000
 -3.012000 1.543000 1.902000
 -1.956000 0.253000 2.490000
 -4.286000 -0.854000 2.951000
 -4.625000 -2.081000 1.715000
```

```
 -3.009000 -1.975000 2.436000
 -4.296000 0.146000 0.638000
 -5.540000 -1.699000 -2.086000
 -5.610000 -1.531000 -0.315000
 -5.258000 -0.120000 -1.325000
 -1.460000 -3.376000 -0.796000
 0.991000 -3.633000 -0.715000
 3.312000 0.222000 -0.656000
 5.782000 -0.132000 -0.499000
 2.813000 -3.808000 0.763000
!entry.L4.unit.residueconnect table  int c1x  int c2x  int c3x  int c4x  int c5x  int c6x
 0 0 0 0 0 0
!entry.L4.unit.residues table  str name  int seq  int childseq  int startatomx  str restype  int
imagingx
 "L4" 1 51 1 "?" 0
!entry.L4.unit.residuesPdbSequenceNumber array int
 0
!entry.L4.unit.solventcap array dbl
 -1.000000
 0.0
 0.0
 0.0
 0.0
!entry.L4.unit.velocities table  dbl x  dbl y  dbl z
 0.0 0.0 0.0
 0.0 0.0 0.0
 0.0 0.0 0.0
 0.0 0.0 0.0
 0.0 0.0 0.0
 0.0 0.0 0.0
 0.0 0.0 0.0
 0.0 0.0 0.0
 0.0 0.0 0.0
 0.0 0.0 0.0
 0.0 0.0 0.0
 0.0 0.0 0.0
 0.0 0.0 0.0
 0.0 0.0 0.0
 0.0 0.0 0.0
 0.0 0.0 0.0
 0.0 0.0 0.0
 0.0 0.0 0.0
 0.0 0.0 0.0
 0.0 0.0 0.0
 0.0 0.0 0.0
 0.0 0.0 0.0
 0.0 0.0 0.0
 0.0 0.0 0.0
 0.0 0.0 0.0
 0.0 0.0 0.0
 0.0 0.0 0.0
 0.0 0.0 0.0
 0.0 0.0 0.0
 0.0 0.0 0.0
 0.0 0.0 0.0
 0.0 0.0 0.0
 0.0 0.0 0.0
 0.0 0.0 0.0
 0.0 0.0 0.0
 0.0 0.0 0.0
 0.0 0.0 0.0
 0.0 0.0 0.0
 0.0 0.0 0.0
 0.0 0.0 0.0
 0.0 0.0 0.0
 0.0 0.0 0.0
 0.0 0.0 0.0
 0.0 0.0 0.0
 0.0 0.0 0.0
 0.0 0.0 0.0
 0.0 0.0 0.0
```

```
 0.0 0.0 0.0
 0.0 0.0 0.0
 0.0 0.0 0.0


!!index array str
 "BEN"
!entry.BEN.unit.atoms table  str name  str type  int typex  int resx  int flags  int seq  int 
elmnt  dbl chg
 "C1" "ca" 0 1 131072 1 6 -0.130000
 "C2" "ca" 0 1 131072 2 6 -0.130000
 "C3" "ca" 0 1 131072 3 6 -0.130000
 "C4" "ca" 0 1 131072 4 6 -0.130000
 "C5" "ca" 0 1 131072 5 6 -0.130000
 "C6" "ca" 0 1 131072 6 6 -0.130000
 "H1" "ha" 0 1 131072 7 1 0.130000
 "H2" "ha" 0 1 131072 8 1 0.130000
 "H3" "ha" 0 1 131072 9 1 0.130000
 "H4" "ha" 0 1 131072 10 1 0.130000
 "H5" "ha" 0 1 131072 11 1 0.130000
 "H6" "ha" 0 1 131072 12 1 0.130000
!entry.BEN.unit.atomspertinfo table  str pname  str ptype  int ptypex  int pelmnt  dbl pchg
 "C1" "ca" 0 -1 0.0
 "C2" "ca" 0 -1 0.0
 "C3" "ca" 0 -1 0.0
 "C4" "ca" 0 -1 0.0
 "C5" "ca" 0 -1 0.0
 "C6" "ca" 0 -1 0.0
 "H1" "ha" 0 -1 0.0
 "H2" "ha" 0 -1 0.0
 "H3" "ha" 0 -1 0.0
 "H4" "ha" 0 -1 0.0
 "H5" "ha" 0 -1 0.0
 "H6" "ha" 0 -1 0.0
!entry.BEN.unit.boundbox array dbl
 -1.000000
 0.0
 0.0
 0.0
 0.0
!entry.BEN.unit.childsequence single int
 2
!entry.BEN.unit.connect array int
 0
 0
!entry.BEN.unit.connectivity table  int atom1x  int atom2x  int flags
 1 2 4
 1 6 4
 1 8 1
 2 3 4
 2 9 1
 3 4 4
 3 10 1
 4 5 4
 4 11 1
 5 6 4
 5 12 1
 6 7 1
!entry.BEN.unit.hierarchy table  str abovetype  int abovex  str belowtype  int belowx
 "U" 0 "R" 1
 "R" 1 "A" 1
 "R" 1 "A" 2
 "R" 1 "A" 3
 "R" 1 "A" 4
 "R" 1 "A" 5
 "R" 1 "A" 6
 "R" 1 "A" 7
 "R" 1 "A" 8
 "R" 1 "A" 9
 "R" 1 "A" 10
```

```
 "R" 1 "A" 11
 "R" 1 "A" 12
!entry.BEN.unit.name single str
 "BEN"
!entry.BEN.unit.positions table  dbl x  dbl y  dbl z
 1.117000 0.839000 0.0
 1.285000 -0.547000 0.0
 0.168000 -1.386000 0.0
 -1.116000 -0.839000 0.0
 -1.285000 0.547000 0.0
 -0.168000 1.386000 0.0
 -0.299000 2.466000 -0.001000
 1.986000 1.492000 0.0
 2.285000 -0.973000 0.0
 0.299000 -2.465000 0.0
 -1.986000 -1.492000 0.0
 -2.285000 0.973000 0.0
!entry.BEN.unit.residueconnect table  int c1x  int c2x  int c3x  int c4x  int c5x  int c6x
 0 0 0 0 0 0
!entry.BEN.unit.residues table  str name  int seq  int childseq  int startatomx  str restype  int
imagingx
 "BEN" 1 13 1 "?" 0
!entry.BEN.unit.residuesPdbSequenceNumber array int
 0
!entry.BEN.unit.solventcap array dbl
 -1.000000
 0.0
 0.0
 0.0
 0.0
!entry.BEN.unit.velocities table  dbl x  dbl y  dbl z
 0.0 0.0 0.0
 0.0 0.0 0.0
 0.0 0.0 0.0
 0.0 0.0 0.0
 0.0 0.0 0.0
 0.0 0.0 0.0
 0.0 0.0 0.0
 0.0 0.0 0.0
 0.0 0.0 0.0
 0.0 0.0 0.0
 0.0 0.0 0.0
 0.0 0.0 0.0


!!index array str
 "BZF"
!entry.BZF.unit.atoms table  str name  str type  int typex  int resx  int flags  int seq  int
elmnt  dbl chg
 "C1" "ca" 0 1 131072 1 6 -0.113000
 "C2" "ca" 0 1 131072 2 6 -0.149000
 "C3" "ca" 0 1 131072 3 6 -0.083000
 "C4" "ca" 0 1 131072 4 6 -0.106800
 "C5" "ca" 0 1 131072 5 6 0.058100
 "C6" "ca" 0 1 131072 6 6 -0.121000
 "C7" "cc" 0 1 131072 7 6 -0.177200
 "C8" "cd" 0 1 131072 8 6 -0.014900
 "O1" "os" 0 1 131072 9 8 -0.204200
 "H1" "ha" 0 1 131072 10 1 0.135000
 "H2" "ha" 0 1 131072 11 1 0.133000
 "H3" "ha" 0 1 131072 12 1 0.138000
 "H4" "ha" 0 1 131072 13 1 0.150000
 "H5" "ha" 0 1 131072 14 1 0.165000
 "H6" "h4" 0 1 131072 15 1 0.189000
!entry.BZF.unit.atomspertinfo table  str pname  str ptype  int ptypex  int pelmnt  dbl pchg
 "C1" "ca" 0 -1 0.0
 "C2" "ca" 0 -1 0.0
 "C3" "ca" 0 -1 0.0
 "C4" "ca" 0 -1 0.0
 "C5" "ca" 0 -1 0.0
 "C6" "ca" 0 -1 0.0
```

```
 "C7" "cc" 0 -1 0.0
 "C8" "cd" 0 -1 0.0
 "O1" "os" 0 -1 0.0
 "H1" "ha" 0 -1 0.0
 "H2" "ha" 0 -1 0.0
 "H3" "ha" 0 -1 0.0
 "H4" "ha" 0 -1 0.0
 "H5" "ha" 0 -1 0.0
 "H6" "h4" 0 -1 0.0
!entry.BZF.unit.boundbox array dbl
 -1.000000
 0.0
 0.0
 0.0
 0.0
!entry.BZF.unit.childsequence single int
 2
!entry.BZF.unit.connect array int
 0
 0
!entry.BZF.unit.connectivity table  int atom1x  int atom2x  int flags
 1 2 4
 1 6 4
 1 10 1
 2 3 4
 2 11 1
 3 4 4
 3 12 1
 4 5 4
 4 7 1
 5 6 4
 5 9 1
 6 13 1
 7 8 2
 7 14 1
 8 9 1
 8 15 1
!entry.BZF.unit.hierarchy table  str abovetype  int abovex  str belowtype  int belowx
 "U" 0 "R" 1
 "R" 1 "A" 1
 "R" 1 "A" 2
 "R" 1 "A" 3
 "R" 1 "A" 4
 "R" 1 "A" 5
 "R" 1 "A" 6
 "R" 1 "A" 7
 "R" 1 "A" 8
 "R" 1 "A" 9
 "R" 1 "A" 10
 "R" 1 "A" 11
 "R" 1 "A" 12
 "R" 1 "A" 13
 "R" 1 "A" 14
 "R" 1 "A" 15
!entry.BZF.unit.name single str
 "BZF"
!entry.BZF.unit.positions table  dbl x  dbl y  dbl z
 2.115000 -0.716000 0.0
 2.142000 0.691000 0.0
 0.968000 1.439000 0.0
 -0.258000 0.757000 0.0
 -0.251000 -0.651000 0.0
 0.909000 -1.416000 0.0
 -1.653000 1.131000 0.0
 -2.352000 -0.030000 0.0
 -1.533000 -1.133000 0.0
 3.050000 -1.268000 0.0
 3.101000 1.202000 0.0
 1.002000 2.525000 0.0
 0.870000 -2.501000 0.0
 -2.069000 2.129000 0.0
```

```
 -3.410000 -0.249000 0.0
!entry.BZF.unit.residueconnect table  int c1x  int c2x  int c3x  int c4x  int c5x  int c6x
 0 0 0 0 0 0
!entry.BZF.unit.residues table  str name  int seq  int childseq  int startatomx  str restype  int
imagingx
 "BZF" 1 16 1 "?" 0
!entry.BZF.unit.residuesPdbSequenceNumber array int
 0
!entry.BZF.unit.solventcap array dbl
 -1.000000
 0.0
 0.0
 0.0
 0.0
!entry.BZF.unit.velocities table  dbl x  dbl y  dbl z
 0.0 0.0 0.0
 0.0 0.0 0.0
 0.0 0.0 0.0
 0.0 0.0 0.0
 0.0 0.0 0.0
 0.0 0.0 0.0
 0.0 0.0 0.0
 0.0 0.0 0.0
 0.0 0.0 0.0
 0.0 0.0 0.0
 0.0 0.0 0.0
 0.0 0.0 0.0
 0.0 0.0 0.0
 0.0 0.0 0.0
 0.0 0.0 0.0
```

```
!!index array str
 "ETH"
!entry.ETH.unit.atoms table  str name  str type  int typex  int resx  int flags  int seq  int
elmnt  dbl chg
 "C1" "ca" 0 1 131072 1 6 -0.128000
 "C2" "ca" 0 1 131072 2 6 -0.128000
 "C3" "ca" 0 1 131072 3 6 -0.133000
 "C4" "ca" 0 1 131072 4 6 -0.128000
 "C5" "ca" 0 1 131072 5 6 -0.128000
 "C6" "ca" 0 1 131072 6 6 -0.077300
 "C7" "c3" 0 1 131072 7 6 -0.038100
 "C8" "c3" 0 1 131072 8 6 -0.089100
 "H1" "ha" 0 1 131072 9 1 0.130000
 "H2" "ha" 0 1 131072 10 1 0.130000
 "H3" "ha" 0 1 131072 11 1 0.130000
 "H4" "ha" 0 1 131072 12 1 0.130000
 "H5" "ha" 0 1 131072 13 1 0.130000
 "H6" "hc" 0 1 131072 14 1 0.045700
 "H7" "hc" 0 1 131072 15 1 0.045700
 "H8" "hc" 0 1 131072 16 1 0.036033
 "H9" "hc" 0 1 131072 17 1 0.036033
 "H10" "hc" 0 1 131072 18 1 0.036033
!entry.ETH.unit.atomspertinfo table  str pname  str ptype  int ptypex  int pelmnt  dbl pchg
 "C1" "ca" 0 -1 0.0
 "C2" "ca" 0 -1 0.0
 "C3" "ca" 0 -1 0.0
 "C4" "ca" 0 -1 0.0
 "C5" "ca" 0 -1 0.0
 "C6" "ca" 0 -1 0.0
 "C7" "c3" 0 -1 0.0
 "C8" "c3" 0 -1 0.0
 "H1" "ha" 0 -1 0.0
 "H2" "ha" 0 -1 0.0
 "H3" "ha" 0 -1 0.0
 "H4" "ha" 0 -1 0.0
 "H5" "ha" 0 -1 0.0
 "H6" "hc" 0 -1 0.0
 "H7" "hc" 0 -1 0.0
```

```
 "H8" "hc" 0 -1 0.0
 "H9" "hc" 0 -1 0.0
 "H10" "hc" 0 -1 0.0
!entry.ETH.unit.boundbox array dbl
 -1.000000
 0.0
 0.0
 0.0
 0.0
!entry.ETH.unit.childsequence single int
 2
!entry.ETH.unit.connect array int
 0
 0
!entry.ETH.unit.connectivity table  int atom1x  int atom2x  int flags
 1 2 4
 1 6 4
 1 9 1
 2 3 4
 2 10 1
 3 4 4
 3 11 1
 4 5 4
 4 12 1
 5 6 4
 5 13 1
 6 7 1
 7 8 1
 7 14 1
 7 15 1
 8 16 1
 8 17 1
 8 18 1
!entry.ETH.unit.hierarchy table  str abovetype  int abovex  str belowtype  int belowx
 "U" 0 "R" 1
 "R" 1 "A" 1
 "R" 1 "A" 2
 "R" 1 "A" 3
 "R" 1 "A" 4
 "R" 1 "A" 5
 "R" 1 "A" 6
 "R" 1 "A" 7
 "R" 1 "A" 8
 "R" 1 "A" 9
 "R" 1 "A" 10
 "R" 1 "A" 11
 "R" 1 "A" 12
 "R" 1 "A" 13
 "R" 1 "A" 14
 "R" 1 "A" 15
 "R" 1 "A" 16
 "R" 1 "A" 17
 "R" 1 "A" 18
!entry.ETH.unit.name single str
 "ETH"
!entry.ETH.unit.positions table  dbl x  dbl y  dbl z
 -0.270000 -1.203000 -0.184000
 -1.638000 -1.206000 0.096000
 -2.326000 0.0 0.237000
 -1.637000 1.206000 0.096000
 -0.270000 1.203000 -0.184000
 0.435000 0.0 -0.328000
 1.926000 0.0 -0.592000
 2.763000 0.0 0.700000
 0.258000 -2.147000 -0.298000
 -2.166000 -2.150000 0.199000
 -3.392000 0.0 0.452000
 -2.165000 2.151000 0.198000
 0.258000 2.147000 -0.298000
 2.192000 0.879000 -1.193000
 2.192000 -0.880000 -1.192000
```

```
 2.544000 0.885000 1.309000
 3.836000 0.0 0.472000
 2.545000 -0.884000 1.309000
!entry.ETH.unit.residueconnect table  int c1x  int c2x  int c3x  int c4x  int c5x  int c6x
 0 0 0 0 0 0
!entry.ETH.unit.residues table  str name  int seq  int childseq  int startatomx  str restype  int
imagingx
 "ETH" 1 19 1 "?" 0
!entry.ETH.unit.residuesPdbSequenceNumber array int
 0
!entry.ETH.unit.solventcap array dbl
 -1.000000
 0.0
 0.0
 0.0
 0.0
!entry.ETH.unit.velocities table  dbl x  dbl y  dbl z
 0.0 0.0 0.0
 0.0 0.0 0.0
 0.0 0.0 0.0
 0.0 0.0 0.0
 0.0 0.0 0.0
 0.0 0.0 0.0
 0.0 0.0 0.0
 0.0 0.0 0.0
 0.0 0.0 0.0
 0.0 0.0 0.0
 0.0 0.0 0.0
 0.0 0.0 0.0
 0.0 0.0 0.0
 0.0 0.0 0.0
 0.0 0.0 0.0
 0.0 0.0 0.0
 0.0 0.0 0.0
 0.0 0.0 0.0
```

```
!!index array str
 "IDE"
!entry.IDE.unit.atoms table  str name  str type  int typex  int resx  int flags  int seq  int
elmnt  dbl chg
 "C1" "ca" 0 1 131072 1 6 -0.129000
 "C2" "ca" 0 1 131072 2 6 -0.133000
 "C3" "ca" 0 1 131072 3 6 -0.107000
 "C4" "ca" 0 1 131072 4 6 -0.071800
 "C5" "ca" 0 1 131072 5 6 -0.094300
 "C6" "ca" 0 1 131072 6 6 -0.115000
 "C7" "ce" 0 1 131072 7 6 -0.117200
 "C8" "c2" 0 1 131072 8 6 -0.173200
 "C9" "c3" 0 1 131072 9 6 -0.019900
 "H1" "ha" 0 1 131072 10 1 0.130000
 "H2" "ha" 0 1 131072 11 1 0.130000
 "H3" "ha" 0 1 131072 12 1 0.134000
 "H4" "ha" 0 1 131072 13 1 0.132000
 "H5" "ha" 0 1 131072 14 1 0.144000
 "H6" "ha" 0 1 131072 15 1 0.142000
 "H7" "hc" 0 1 131072 16 1 0.074700
 "H8" "hc" 0 1 131072 17 1 0.074700
!entry.IDE.unit.atomspertinfo table  str pname  str ptype  int ptypex  int pelmnt  dbl pchg
 "C1" "ca" 0 -1 0.0
 "C2" "ca" 0 -1 0.0
 "C3" "ca" 0 -1 0.0
 "C4" "ca" 0 -1 0.0
 "C5" "ca" 0 -1 0.0
 "C6" "ca" 0 -1 0.0
 "C7" "ce" 0 -1 0.0
 "C8" "c2" 0 -1 0.0
 "C9" "c3" 0 -1 0.0
 "H1" "ha" 0 -1 0.0
 "H2" "ha" 0 -1 0.0
```

```
 "H3" "ha" 0 -1 0.0
 "H4" "ha" 0 -1 0.0
 "H5" "ha" 0 -1 0.0
 "H6" "ha" 0 -1 0.0
 "H7" "hc" 0 -1 0.0
 "H8" "hc" 0 -1 0.0
!entry.IDE.unit.boundbox array dbl
 -1.000000
 0.0
 0.0
 0.0
 0.0
!entry.IDE.unit.childsequence single int
 2
!entry.IDE.unit.connect array int
 0
 0
!entry.IDE.unit.connectivity table  int atom1x  int atom2x  int flags
 1 2 4
 1 6 4
 1 10 1
 2 3 4
 2 11 1
 3 4 4
 3 12 1
 4 5 4
 4 7 1
 5 6 4
 5 9 1
 6 13 1
 7 8 2
 7 14 1
 8 9 1
 8 15 1
 9 16 1
 9 17 1
!entry.IDE.unit.hierarchy table  str abovetype  int abovex  str belowtype  int belowx
 "U" 0 "R" 1
 "R" 1 "A" 1
 "R" 1 "A" 2
 "R" 1 "A" 3
 "R" 1 "A" 4
 "R" 1 "A" 5
 "R" 1 "A" 6
 "R" 1 "A" 7
 "R" 1 "A" 8
 "R" 1 "A" 9
 "R" 1 "A" 10
 "R" 1 "A" 11
 "R" 1 "A" 12
 "R" 1 "A" 13
 "R" 1 "A" 14
 "R" 1 "A" 15
 "R" 1 "A" 16
 "R" 1 "A" 17
!entry.IDE.unit.name single str
 "IDE"
!entry.IDE.unit.positions table  dbl x  dbl y  dbl z
 -2.172000 -0.714000 0.0
 -2.191000 0.684000 0.0
 -1.000000 1.416000 0.0
 0.212000 0.723000 0.0
 0.230000 -0.690000 0.0
 -0.956000 -1.411000 0.0
 1.600000 1.197000 0.0
 2.441000 0.144000 0.0
 1.670000 -1.156000 0.0
 -3.109000 -1.265000 0.0
 -3.144000 1.208000 0.0
 -1.020000 2.503000 0.0
 -0.950000 -2.499000 0.0
```

```
 1.886000 2.244000 0.0
 3.524000 0.192000 0.0
 1.908000 -1.774000 -0.878000
 1.908000 -1.773000 0.879000
!entry.IDE.unit.residueconnect table  int c1x  int c2x  int c3x  int c4x  int c5x  int c6x
 0 0 0 0 0 0
!entry.IDE.unit.residues table  str name  int seq  int childseq  int startatomx  str restype  int
imagingx
 "IDE" 1 18 1 "?" 0
!entry.IDE.unit.residuesPdbSequenceNumber array int
 0
!entry.IDE.unit.solventcap array dbl
 -1.000000
 0.0
 0.0
 0.0
 0.0
!entry.IDE.unit.velocities table  dbl x  dbl y  dbl z
 0.0 0.0 0.0
 0.0 0.0 0.0
 0.0 0.0 0.0
 0.0 0.0 0.0
 0.0 0.0 0.0
 0.0 0.0 0.0
 0.0 0.0 0.0
 0.0 0.0 0.0
 0.0 0.0 0.0
 0.0 0.0 0.0
 0.0 0.0 0.0
 0.0 0.0 0.0
 0.0 0.0 0.0
 0.0 0.0 0.0
 0.0 0.0 0.0
 0.0 0.0 0.0
 0.0 0.0 0.0


!!index array str
 "IDO"
!entry.IDO.unit.atoms table  str name  str type  int typex  int resx  int flags  int seq  int
elmnt  dbl chg
 "C1" "ca" 0 1 131072 1 6 -0.113000
 "C2" "ca" 0 1 131072 2 6 -0.159000
 "C3" "ca" 0 1 131072 3 6 -0.082000
 "C4" "ca" 0 1 131072 4 6 -0.095800
 "C5" "ca" 0 1 131072 5 6 -0.047200
 "C6" "ca" 0 1 131072 6 6 -0.146000
 "C7" "cc" 0 1 131072 7 6 -0.187200
 "C8" "cd" 0 1 131072 8 6 -0.109100
 "N1" "na" 0 1 131072 9 7 -0.196400
 "H1" "ha" 0 1 131072 10 1 0.128000
 "H2" "ha" 0 1 131072 11 1 0.128000
 "H3" "ha" 0 1 131072 12 1 0.133000
 "H4" "ha" 0 1 131072 13 1 0.130000
 "H5" "ha" 0 1 131072 14 1 0.156000
 "H6" "h4" 0 1 131072 15 1 0.163000
 "H7" "hn" 0 1 131072 16 1 0.297700
!entry.IDO.unit.atomspertinfo table  str pname  str ptype  int ptypex  int pelmnt  dbl pchg
 "C1" "ca" 0 -1 0.0
 "C2" "ca" 0 -1 0.0
 "C3" "ca" 0 -1 0.0
 "C4" "ca" 0 -1 0.0
 "C5" "ca" 0 -1 0.0
 "C6" "ca" 0 -1 0.0
 "C7" "cc" 0 -1 0.0
 "C8" "cd" 0 -1 0.0
 "N1" "na" 0 -1 0.0
 "H1" "ha" 0 -1 0.0
 "H2" "ha" 0 -1 0.0
 "H3" "ha" 0 -1 0.0
```

```
 "H4" "ha" 0 -1 0.0
 "H5" "ha" 0 -1 0.0
 "H6" "h4" 0 -1 0.0
 "H7" "hn" 0 -1 0.0
!entry.IDO.unit.boundbox array dbl
 -1.000000
 0.0
 0.0
 0.0
 0.0
!entry.IDO.unit.childsequence single int
 2
!entry.IDO.unit.connect array int
 0
 0
!entry.IDO.unit.connectivity table  int atom1x  int atom2x  int flags
 1 2 4
 1 6 4
 1 10 1
 2 3 4
 2 11 1
 3 4 4
 3 12 1
 4 5 4
 4 7 1
 5 6 4
 5 9 1
 6 13 1
 7 8 2
 7 14 1
 8 9 1
 8 15 1
 9 16 1
!entry.IDO.unit.hierarchy table  str abovetype  int abovex  str belowtype  int belowx
 "U" 0 "R" 1
 "R" 1 "A" 1
 "R" 1 "A" 2
 "R" 1 "A" 3
 "R" 1 "A" 4
 "R" 1 "A" 5
 "R" 1 "A" 6
 "R" 1 "A" 7
 "R" 1 "A" 8
 "R" 1 "A" 9
 "R" 1 "A" 10
 "R" 1 "A" 11
 "R" 1 "A" 12
 "R" 1 "A" 13
 "R" 1 "A" 14
 "R" 1 "A" 15
 "R" 1 "A" 16
!entry.IDO.unit.name single str
 "IDO"
!entry.IDO.unit.positions table  dbl x  dbl y  dbl z
 2.136000 -0.719000 0.0
 2.159000 0.692000 0.0
 0.982000 1.429000 0.0
 -0.250000 0.752000 0.0
 -0.248000 -0.672000 0.0
 0.935000 -1.419000 0.0
 -1.626000 1.167000 0.0
 -2.390000 0.030000 0.0
 -1.566000 -1.081000 0.0
 3.074000 -1.268000 0.0
 3.116000 1.206000 0.0
 1.011000 2.516000 0.0
 0.918000 -2.506000 0.0
 -1.999000 2.182000 0.0
 -3.465000 -0.087000 0.0
 -1.881000 -2.039000 -0.001000
!entry.IDO.unit.residueconnect table  int c1x  int c2x  int c3x  int c4x  int c5x  int c6x
```

```
 0 0 0 0 0 0
!entry.IDO.unit.residues table  str name  int seq  int childseq  int startatomx  str restype  int imagingx
 "IDO" 1 17 1 "?" 0
!entry.IDO.unit.residuesPdbSequenceNumber array int
 0
!entry.IDO.unit.solventcap array dbl
 -1.000000
 0.0
 0.0
 0.0
 0.0
!entry.IDO.unit.velocities table  dbl x  dbl y  dbl z
 0.0 0.0 0.0
 0.0 0.0 0.0
 0.0 0.0 0.0
 0.0 0.0 0.0
 0.0 0.0 0.0
 0.0 0.0 0.0
 0.0 0.0 0.0
 0.0 0.0 0.0
 0.0 0.0 0.0
 0.0 0.0 0.0
 0.0 0.0 0.0
 0.0 0.0 0.0
 0.0 0.0 0.0
 0.0 0.0 0.0
 0.0 0.0 0.0
 0.0 0.0 0.0
```

```
!!index array str
 "PHN"
!entry.PHN.unit.atoms table  str name  str type  int typex  int resx  int flags  int seq  int elmnt  dbl chg
 "C1" "ca" 0 1 131072 1 6 -0.319049
 "H1" "ha" 0 1 131072 2 1 0.175048
 "C2" "ca" 0 1 131072 3 6 -0.077264
 "H2" "ha" 0 1 131072 4 1 0.138265
 "C3" "ca" 0 1 131072 5 6 -0.216517
 "H3" "ha" 0 1 131072 6 1 0.143384
 "C4" "ca" 0 1 131072 7 6 -0.077264
 "H4" "ha" 0 1 131072 8 1 0.138265
 "C5" "ca" 0 1 131072 9 6 -0.319049
 "H5" "ha" 0 1 131072 10 1 0.175048
 "C6" "ca" 0 1 131072 11 6 0.413994
 "O1" "oh" 0 1 131072 12 8 -0.553947
 "H6" "ho" 0 1 131072 13 1 0.379087
!entry.PHN.unit.atomspertinfo table  str pname  str ptype  int ptypex  int pelmnt  dbl pchg
 "C1" "ca" 0 -1 0.0
 "H1" "ha" 0 -1 0.0
 "C2" "ca" 0 -1 0.0
 "H2" "ha" 0 -1 0.0
 "C3" "ca" 0 -1 0.0
 "H3" "ha" 0 -1 0.0
 "C4" "ca" 0 -1 0.0
 "H4" "ha" 0 -1 0.0
 "C5" "ca" 0 -1 0.0
 "H5" "ha" 0 -1 0.0
 "C6" "ca" 0 -1 0.0
 "O1" "oh" 0 -1 0.0
 "H6" "ho" 0 -1 0.0
!entry.PHN.unit.boundbox array dbl
 -1.000000
 0.0
 0.0
 0.0
 0.0
!entry.PHN.unit.childsequence single int
 2
```

```
!entry.PHN.unit.connect array int
 1
 12
!entry.PHN.unit.connectivity table  int atom1x  int atom2x  int flags
 1 2 1
 1 3 1
 1 11 1
 3 4 1
 3 5 1
 5 6 1
 5 7 1
 7 8 1
 7 9 1
 9 10 1
 9 11 1
 11 12 1
 12 13 1
!entry.PHN.unit.hierarchy table  str abovetype  int abovex  str belowtype  int belowx
 "U" 0 "R" 1
 "R" 1 "A" 1
 "R" 1 "A" 2
 "R" 1 "A" 3
 "R" 1 "A" 4
 "R" 1 "A" 5
 "R" 1 "A" 6
 "R" 1 "A" 7
 "R" 1 "A" 8
 "R" 1 "A" 9
 "R" 1 "A" 10
 "R" 1 "A" 11
 "R" 1 "A" 12
 "R" 1 "A" 13
!entry.PHN.unit.name single str
 "PHN"
!entry.PHN.unit.positions table  dbl x  dbl y  dbl z
 0.265000 1.189000 0.0
 0.820000 2.112000 0.0
 -1.122000 1.209000 0.0
 -1.634000 2.155000 0.0
 -1.844000 0.030000 0.0
 -2.919000 0.049000 0.0
 -1.162000 -1.180000 0.0
 -1.711000 -2.105000 0.0
 0.219000 -1.215000 0.0
 0.757000 -2.144000 0.0
 0.934000 -0.024000 0.0
 2.284000 -0.109000 -0.001000
 2.672000 0.755000 0.009000
!entry.PHN.unit.residueconnect table  int c1x  int c2x  int c3x  int c4x  int c5x  int c6x
 1 12 0 0 0 0
!entry.PHN.unit.residues table  str name  int seq  int childseq  int startatomx  str restype  int
imagingx
 "PHN" 1 14 1 "?" 0
!entry.PHN.unit.residuesPdbSequenceNumber array int
 0
!entry.PHN.unit.solventcap array dbl
 -1.000000
 0.0
 0.0
 0.0
 0.0
!entry.PHN.unit.velocities table  dbl x  dbl y  dbl z
 0.0 0.0 0.0
 0.0 0.0 0.0
 0.0 0.0 0.0
 0.0 0.0 0.0
 0.0 0.0 0.0
 0.0 0.0 0.0
 0.0 0.0 0.0
 0.0 0.0 0.0
 0.0 0.0 0.0
```

```
 0.0 0.0 0.0
 0.0 0.0 0.0
 0.0 0.0 0.0
 0.0 0.0 0.0
```

```
!!index array str
 "TOL"
!entry.TOL.unit.atoms table  str name  str type  int typex  int resx  int flags  int seq  int
elmnt  dbl chg
 "C1" "ca" 0 1 131072 1 6 -0.131000
 "C2" "ca" 0 1 131072 2 6 -0.127000
 "C3" "ca" 0 1 131072 3 6 -0.135000
 "C4" "ca" 0 1 131072 4 6 -0.127000
 "C5" "ca" 0 1 131072 5 6 -0.131000
 "C6" "ca" 0 1 131072 6 6 -0.077300
 "C7" "c3" 0 1 131072 7 6 -0.053800
 "H1" "hc" 0 1 131072 8 1 0.044033
 "H2" "ha" 0 1 131072 9 1 0.130000
 "H3" "ha" 0 1 131072 10 1 0.130000
 "H4" "ha" 0 1 131072 11 1 0.130000
 "H5" "ha" 0 1 131072 12 1 0.130000
 "H6" "ha" 0 1 131072 13 1 0.130000
 "H7" "hc" 0 1 131072 14 1 0.044033
 "H8" "hc" 0 1 131072 15 1 0.044033
!entry.TOL.unit.atomspertinfo table  str pname  str ptype  int ptypex  int pelmnt  dbl pchg
 "C1" "ca" 0 -1 0.0
 "C2" "ca" 0 -1 0.0
 "C3" "ca" 0 -1 0.0
 "C4" "ca" 0 -1 0.0
 "C5" "ca" 0 -1 0.0
 "C6" "ca" 0 -1 0.0
 "C7" "c3" 0 -1 0.0
 "H1" "hc" 0 -1 0.0
 "H2" "ha" 0 -1 0.0
 "H3" "ha" 0 -1 0.0
 "H4" "ha" 0 -1 0.0
 "H5" "ha" 0 -1 0.0
 "H6" "ha" 0 -1 0.0
 "H7" "hc" 0 -1 0.0
 "H8" "hc" 0 -1 0.0
!entry.TOL.unit.boundbox array dbl
 -1.000000
 0.0
 0.0
 0.0
 0.0
!entry.TOL.unit.childsequence single int
 2
!entry.TOL.unit.connect array int
 0
 0
!entry.TOL.unit.connectivity table  int atom1x  int atom2x  int flags
 1 2 4
 1 6 4
 1 9 1
 2 3 4
 2 10 1
 3 4 4
 3 11 1
 4 5 4
 4 12 1
 5 6 4
 5 13 1
 6 7 1
 7 8 1
 7 14 1
 7 15 1
!entry.TOL.unit.hierarchy table  str abovetype  int abovex  str belowtype  int belowx
 "U" 0 "R" 1
```

```
 "R" 1 "A" 1
 "R" 1 "A" 2
 "R" 1 "A" 3
 "R" 1 "A" 4
 "R" 1 "A" 5
 "R" 1 "A" 6
 "R" 1 "A" 7
 "R" 1 "A" 8
 "R" 1 "A" 9
 "R" 1 "A" 10
 "R" 1 "A" 11
 "R" 1 "A" 12
 "R" 1 "A" 13
 "R" 1 "A" 14
 "R" 1 "A" 15
!entry.TOL.unit.name single str
 "TOL"
!entry.TOL.unit.positions table  dbl x  dbl y  dbl z
 0.194000 1.202000 -0.009000
 -1.201000 1.205000 0.002000
 -1.905000 0.0 0.008000
 -1.201000 -1.205000 0.002000
 0.194000 -1.202000 -0.009000
 0.914000 0.0 -0.011000
 2.425000 0.0 0.009000
 2.834000 -0.889000 -0.484000
 0.735000 2.147000 -0.018000
 -1.738000 2.151000 0.001000
 -2.992000 0.0 0.014000
 -1.739000 -2.150000 0.001000
 0.734000 -2.147000 -0.018000
 2.810000 0.006000 1.038000
 2.834000 0.883000 -0.493000
!entry.TOL.unit.residueconnect table  int c1x  int c2x  int c3x  int c4x  int c5x  int c6x
 0 0 0 0 0 0
!entry.TOL.unit.residues table  str name  int seq  int childseq  int startatomx  str restype  int
imagingx
 "TOL" 1 16 1 "?" 0
!entry.TOL.unit.residuesPdbSequenceNumber array int
 0
!entry.TOL.unit.solventcap array dbl
 -1.000000
 0.0
 0.0
 0.0
 0.0
!entry.TOL.unit.velocities table  dbl x  dbl y  dbl z
 0.0 0.0 0.0
 0.0 0.0 0.0
 0.0 0.0 0.0
 0.0 0.0 0.0
 0.0 0.0 0.0
 0.0 0.0 0.0
 0.0 0.0 0.0
 0.0 0.0 0.0
 0.0 0.0 0.0
 0.0 0.0 0.0
 0.0 0.0 0.0
 0.0 0.0 0.0
 0.0 0.0 0.0
 0.0 0.0 0.0
 0.0 0.0 0.0
```

Table S9. AMBER additional parameters for the lysozyme ligands (frcmod file).

```
Non-standard parameters for the lysozyme ligands
MASS

BOND

ANGLE

DIHE
ca-ca-cc-cd   4    2.800       180.000           2.000      same as X -c2-ca-X , penalty
score=232.0
ca-ca-cc-ha   4    2.800       180.000           2.000      same as X -c2-ca-X , penalty
score=232.0
ca-ca-ce-c2   4    2.800       180.000           2.000      same as X -c2-ca-X , penalty
score=237.0
ca-ca-ce-ha   4    2.800       180.000           2.000      same as X -c2-ca-X , penalty
score=237.0
cc-cd-os-ca   2    2.100       180.000           2.000      same as X -c2-os-X , penalty
score=232.0
h4-cd-os-ca   2    2.100       180.000           2.000      same as X -c2-os-X , penalty
score=232.0

IMPROPER
ca-ca-ca-cc         1.1          180.0         2.0          Using the default value
ca-ca-ca-ce         1.1          180.0         2.0          Using the default value
ca-ca-ca-ha         1.1          180.0         2.0          Using general improper torsional
angle  X- X-ca-ha, penalty score=  6.0)
ca-ca-ca-na         1.1          180.0         2.0          Using the default value
ca-ca-ca-os         1.1          180.0         2.0          Using the default value
ca-cd-cc-ha         1.1          180.0         2.0          Same as X -X -ca-ha, penalty score=
38.9 (use general term))
ca-cd-na-hn         1.1          180.0         2.0          Using general improper torsional
angle  X- X-na-hn, penalty score=  6.0)
cc-h4-cd-na         1.1          180.0         2.0          Same as X -X -ca-ha, penalty score=
67.2 (use general term))
cc-h4-cd-os         1.1          180.0         2.0          Same as X -X -ca-ha, penalty score=
67.2 (use general term))
c2-ca-ce-ha         1.1          180.0         2.0          Same as X -X -ca-ha, penalty score=
46.8 (use general term))
c3-ce-c2-ha         1.1          180.0         2.0          Same as X -X -ca-ha, penalty score=
47.1 (use general term))

NONBON
```

References

1. P. Mikulskis, S. Genheden and U. Ryde, *J. Chem. Inf. Model.*, 2014, **54**, 2794-2806.
2. G. Dolcetti, V. Ekberg, L. Cao, M. M. Ignjatović, M. A. Olsson and U. Ryde, *J. Chem. Inf. Model*, 2025, submitted.
3. Y. Yuthavong, T. Vilaivan, N. Chareonsethakul, S. Kamchonwongpaisan, W. Sirawaraporn, R. Quarrell and G. Lowe, *Journal of Medicinal Chemistry*, 2000, **43**, 2738-2744.
4. B. Tarnchompoo, C. Sirichaiwat, W. Phupong, C. Intaraudom, W. Sirawaraporn, S. Kamchonwongpaisan, J. Vanichtanankul, Y. Thebtaranonth and Y. Yuthavong, *J Med Chem*, 2002, **45**, 1244-1252.
5. H. Matter, E. Defossa, U. Heinelt, P. M. Blohm, D. Schneider, A. Muller, S. Herok, H. Schreuder, A. Liesum, V. Brachvogel, P. Lonze, A. Walser, F. Al-Obeidi and P. Wildgoose, *J Med Chem*, 2002, **45**, 2749-2769.
6. L. S. Vedula, G. Brannigan, N. J. Economou, J. Xi, M. A. Hall, R. Liu, M. J. Rossi, W. P. Dailey, K. C. Grasty, M. L. Klein, R. G. Eckenhoff and P. J. Loll, *J Biol Chem*, 2009, **284**, 24176-24184.
